# In water or on land? A network meta-analysis of aquatic and land-based exercise interventions for pain and disability in chronic lower back pain

**DOI:** 10.3389/fmed.2026.1739263

**Published:** 2026-02-02

**Authors:** Hao Wu, Penglin Diao, Juan Del Coso, Xiaoyu Liu, Yiwei Min, Ruimeng Ran, Bopeng Qiu, Yinkai Zhang, Ziyu Wang, Carl Petersen

**Affiliations:** 1 Sports Institute, Henan University of Technology, Zhengzhou, China; 2 China Wushu School, Beijing Sport University, Beijing, China; 3 School of Sports Training, Wuhan Sports University, Wuhan, China; 4 Sport Sciences Research Centre, Rey Juan Carlos University, Fuenlabrada, Spain; 5 Faculty of Health Sciences, Institute of Health and Sport Sciences, Universidad Francisco de Vitoria, Madrid, Spain; 6 School of Arts and Sciences, Fuyao University of Science and Technology, Fuzhou, China; 7 Department of Kinesiology and Sport Management, Texas A&M University, College Station, TX, United States; 8 College of Education, Beijing Sport University, Beijing, China; 9 Division of Sports Science and Physical Education, Tsinghua University, Beijing, China; 10 Faculty of Health, University of Canterbury, Christchurch, New Zealand

**Keywords:** aquatic exercise, balneotherapy, chronic low back pain, hydrotherapy, physical therapy, rehabilitation, therapeutic exercise

## Abstract

**Background:**

Chronic non-specific low back pain (CLBP) imposes a substantial healthcare burden. For CLBP, non-pharmacologic pain management within physical therapy/rehabilitation commonly relies on therapeutic exercise (exercise therapy). Aquatic interventions such as exercise and balneotherapy are widely prescribed to treat CLBP, but their comparative effectiveness against land-based exercise, and multi-model programs remain unclear. We performed a network meta-analysis of randomized controlled trials to compare the effects of aquatic, land-based exercise, and multi-modal interventions on pain and disability in patients with chronic low back pain.

**Methods:**

We searched from inception to May 2025 for randomized controlled trials in adults with chronic non-specific low back pain that evaluated water-based therapies (aquatic exercise, hydrotherapy/balneotherapy). All randomized arms within eligible trials were retained, allowing comparisons with land-based exercise, combined aquatic + land-based programs, and general care/blank control.

**Results:**

We included 26 RCTs, forming a 9-node network (blank control, general care, land-based exercise, aquatic exercise, balneotherapy, and their combinations with general care/land-based exercise). For pain intensity, compared with blank control, balneotherapy + general care (SMD = 2.51, 95% CI 1.26–3.76; *p* < 0.001), aquatic exercise + general care (SMD = 1.96, 95% CI 0.44–3.48; *p* = 0.011), balneotherapy + land-based exercise (SMD = 1.68, 95% CI 0.58–2.78; *p* = 0.003), aquatic exercise (SMD = 1.58, 95% CI 0.83–2.32; *p* < 0.001), land-based exercise (SMD = 1.45, 95% CI 0.56–2.34; *p* = 0.001), and balneotherapy (SMD = 1.27, 95% CI 0.19–2.35; *p* = 0.021) significantly reduced pain. By contrast, land-based exercise + general care (SMD = 1.55, 95% CI − 0.18–3.27; *p* = 0.078) and general care (SMD = 1.02, 95% CI − 0.13–2.17; *p* = 0.081) did not reach statistical significance. Based on SUCRA for pain, balneotherapy + general care ranked first (SUCRA = 0.92; PrBest = 61.4%; mean rank = 1.7), followed by aquatic exercise + general care (0.71; 21.5%; 3.3) and balneotherapy + land-based exercise (0.62; 3.8%; 4.0), with combined interventions generally ranking above single modalities; blank control ranked last (0.01; 0.0%; 8.9). For clinical context, the observed SMDs correspond approximately to 24–48 points of pain reduction across the interventions that showed statistically significant effects. For disability, compared with blank control, balneotherapy + general care (SMD = 2.76, 95% CI 1.12–4.40; *p* = 0.001), balneotherapy (SMD = 2.48, 95% CI 0.50–4.45; *p* = 0.014), aquatic exercise + general care (SMD = 2.28, 95% CI 0.69–3.86; *p* = 0.005), balneotherapy + land-based exercise (SMD = 2.06, 95% CI 0.64–3.48; *p* = 0.004), aquatic exercise (SMD = 2.03, 95% CI 1.20–2.87; *p* < 0.001), land-based exercise (SMD = 1.84, 95% CI 0.80–2.88; *p* = 0.001), and general care (SMD = 1.66, 95% CI 0.11–3.21; *p* = 0.035) significantly reduced disability, whereas land-based exercise + general care did not (SMD = 1.72, 95% CI − 0.25–3.68; *p* = 0.086). SUCRA rankings for disability again favored multimodal care: balneotherapy + general care ranked first (SUCRA = 0.83; PrBest = 44.6%; mean rank = 2.4), followed by balneotherapy (0.71; 29.3%; 3.3) and aquatic exercise + general care (0.64; 16.9%; 3.9), with blank control consistently last (0.01; 0.0%; 8.9). Given very high heterogeneity and low-to-very-low certainty for most comparisons, these findings should be interpreted as preliminary directional evidence rather than actionable treatment recommendations. Results suggest multimodal approaches warrant investigation in future rigorously conducted trials.

**Conclusion:**

In CLBP, multimodal programs that integrate hydrotherapeutic components with general care or exercise tended to provide greater improvements than single interventions for both pain and disability. Among single modalities, effects were outcome-specific: for pain, aquatic exercise performed best and land-based exercise generally exceeded balneotherapy; for disability, balneotherapy and aquatic exercise showed larger improvements than land-based exercise. Land-based exercise remains a beneficial and pragmatic option where aquatic access is limited, while adding general care to land-based exercise did not show a consistent additional benefit. This analysis is substantially limited by very high heterogeneity (*I*^2^ > 85%) and predominantly very low certainty evidence. Of the 36 comparisons assessed for pain intensity, 94.4% (34/36) were rated low to very low certainty; for disability, all 36 comparisons were rated very low certainty. These evidence quality profiles severely restrict ranking reliability and recommendation strength.

**Systematic review registration:**

Registered on PROSPERO. Unique Identifier: CRD42023432018. Public URL: https://www.crd.york.ac.uk/PROSPERO/view/CRD42023432018.

## 1 Introduction

Chronic low back pain (CLBP) refers to pain in the lower back that persists for more than 12 weeks, located between the lower ribs and the gluteal fold, and may or may not be accompanied by lower limb pain (1). It is a highly prevalent and increasingly severe musculoskeletal disorder and a leading cause of disability worldwide (2). Approximately 90% of low back pain cases are mild and typically resolve within a few days to several weeks; however, up to 10% progress to CLBP (3). In a US insurance claims analysis, Hashemi et al. reported that up to 8.8% of individuals with low back pain experienced symptoms persisting for 1 year, and this subgroup accounted for as much as 84.7% of the total healthcare costs associated with low back pain treatment (4).

Among available treatments for CLBP, aquatic interventions leverages the unique physical properties of water to promote muscle relaxation, alleviate pain, and improve motor function, emerging as a promising treatment approach (5). While aquatic interventions, including aquatic exercise and hydrotherapy, has demonstrated benefits in pain relief (6), evidence comparing the efficacy of different aquatic intervention modalities remains limited. Previous systematic reviews suggest that aquatic physiotherapy, hydrotherapy, and spa therapy can reduce pain and disability in patients with chronic low back pain (7–9). However, much of this evidence is based on small or methodologically limited trials, and findings for balneotherapy in particular remain inconsistent (8, 9). Moreover, prior meta-analyses have been restricted to pairwise comparisons (7–9), making it difficult to establish the relative effectiveness of aquatic, land-based, and multimodal interventions.

Although current evidence indicates benefits from several aquatic interventions, differences in study design and outcome measures make direct comparisons difficult, limiting their usefulness for clinicians when choosing among available treatments for CLBP (7–9). Specifically, limitations arise at the trial level, where comparisons are predominantly head-to-head and interventions are frequently multi-component, making it difficult to isolate the effects of individual aquatic modalities. Aquatic interventions and control conditions varied widely across trials: some compared mineral thermal baths with tap water baths (10), others used hyperthermic hydrotherapy compared to controls from a waiting lists (11, 12), and several combined bathing with exercise or physiotherapy rather than testing it in isolation (13–17). These inconsistent designs and frequent co-interventions make it difficult to attribute observed effects to specific aquatic or balneotherapy modalities.

These inconsistent control designs and the confounding presence of co-interventions make it difficult to definitively attribute effects to any specific aquatic/balneotherapy modality.

Network meta-analysis (NMA) can overcome these limitations by incorporating data from RCTs that do not necessarily share identical comparison groups within a connected “network” of evidence (18). In an NMA, we can include trials comparing two or more active interventions without requiring a conventional control group (19). This approach enables both direct comparisons (as in pairwise meta-analysis) and indirect comparisons across treatments via the network (20), allowing researchers to rank interventions in terms of their relative effectiveness. Therefore, the aim of this study was to compare aquatic interventions with other available treatment options in adults with CLBP using a network meta-analysis of randomized controlled trials. Importantly, a critical caveat is that this network was constructed through a search targeting aquatic interventions; consequently, evidence for non-aquatic nodes derives exclusively from trials also evaluating aquatic comparisons, which may systematically bias network topology and limit generalizability of non-aquatic comparisons. We hypothesized that multimodal interventions incorporating aquatic therapies would yield the greatest improvements as they provided a more comprehensive approach, and that among single interventions, aquatic exercise and balneotherapy would demonstrate superior effectiveness compared with land-based exercise.

## 2 Methods

This review was conducted in accordance with Preferred Reporting Items for Systematic Reviews and Meta-Analyses for Network Meta-Analyses (PRISMA-NMA) guidelines (21), and the study protocol was registered in PROSPERO (CRD42023432018).

### 2.1 Search strategy

We conducted a concept-based search from database inception to May 2025 to identify randomized controlled trials in adults with chronic non-specific low back pain (CLBP) that included at least one water-based therapy arm—aquatic exercise, hydrotherapy/balneotherapy. For each eligible RCT, all randomized arms were retained, so land-based exercise, combined aquatic + land-based programs, and general care/blank control entered the network as comparators when present.

Searches combined subject headings (MeSH/Emtree) and free-text terms for three concept sets: Population (“low back pain,” CLBP), Intervention family (“aquatic therapy,” “hydrotherapy,” “balneotherapy,” “water-based,” “pool exercise,” “deep-water running”), linked with Boolean operators. Full search strategies for each database (MeSH/Emtree terms, keywords, and filters) are provided in Supplementary Tables S1–S3.

### 2.2 Eligibility criteria

We developed eligibility criteria using the PICOS framework (Population, Intervention, Comparator, Outcomes, and Study design), following Cochrane systematic review guidelines (22).

#### 2.2.1 Inclusion criteria

Studies were eligible if they met all of the following:

- Population—Adults (≥18 years) with chronic non-specific CLBP, typically defined as pain ≥12 weeks.
- Intervention—Trials that included at least one water-based therapy arm, defined as aquatic exercise or hydrotherapy/balneotherapy. For each eligible trial, all randomized arms were retained; therefore, land-based exercise, combined aquatic + land-based programs, and general care/blank control entered the network whenever present as comparators. Accordingly, evidence for non-aquatic nodes is derived from trials that also included an aquatic intervention arm, to allow comparisons within a common clinical and methodological context and improve internal validity.
- Comparators and node definitions—Any randomized comparator arm was eligible. Each arm was mapped to one node using the following operational definitions:
- Blank control (no active treatment): wait-list or no-treatment controls without structured therapeutic content and without exercise or hydrotherapeutic sessions; general care: non-structured clinical management (e.g., advice/education/routine care) without an explicit therapeutic exercise or home-exercise prescription; aquatic exercise: structured exercise programs performed in a pool; classification was based on the presence of therapeutic exercise rather than water composition (tap vs. mineral/thermal); balneotherapy: passive immersion/bath therapy in mineral/thermal/spa waters (including hyperthermic or mineral baths with or without mud or peloid packs), provided that no structured therapeutic exercise was delivered in the same arm; land-based exercise: prescribed therapeutic exercise delivered on land, including supervised clinic-based programs and home-based physiotherapy/home-exercise prescriptions (e.g., lumbar strengthening, stabilization, flexibility, or flexion/extension exercises); combined interventions (prespecified): arms delivering two components together were coded accordingly. The allocation for each of the interventions included in the selected studies was performed by two reviewers independently, with disagreements resolved by consensus.
- Outcomes—At least one of: pain intensity or disability/physical function.
- Study design—Parallel-group randomized controlled trials, peer-reviewed, published in English.

Pain intensity was taken from a Visual Analogue Scale (VAS) or a Numeric Rating Scale (NRS). Disability was taken from the Oswestry Disability Index (ODI) or the Roland–Morris Disability Questionnaire (RMDQ). The primary time point was the post-intervention assessment closest to end of the treatment period, and effect sizes were computed using pre-to-post change scores by extracting baseline and immediate post-intervention data from each study.

#### 2.2.2 Exclusion criteria

Studies were excluded if they: (i) included participants with specific causes of LBP (e.g., pregnancy-related pain, infection, cauda equina syndrome); (ii) recruited pre- or post-surgical patients, or those with recurrent LBP (≥6 months pain-free before re-onset); (iii) were non-randomized, observational, review articles, or lacked full-text publication (e.g., abstracts only).

### 2.3 Data extraction

Following the identification and selection of eligible full-text articles, data were independently extracted by two reviewers using a standardized Excel spreadsheet. All extracted information was double-checked to ensure accuracy and consistency. Discrepancies were resolved through discussion, or, if needed, by consultation with a third reviewer. The following data were extracted from each included study:

First author and year of publication; sample size, mean age, and gender distribution of each group; intervention details for all groups (e.g., type, frequency, duration, setting); pre-to-post intervention change in primary and secondary outcomes (e.g., pain intensity and disability scores); and methodological characteristics.

### 2.4 Quality assessment

Risk of bias was independently assessed by two reviewers (HW and PD) using the Cochrane Risk of Bias tool (RoB) (23), which evaluates selection, performance, detection, attrition, reporting, and other biases. Each domain was rated as low, high, or unclear risk. Random sequence generation (e.g., random number generator) was considered low risk, whereas non-random methods (e.g., date of birth) were considered high risk. Given the nature of exercise interventions, blinding of participants was not feasible and was excluded from the overall risk of bias judgement. Discrepancies were resolved by a third reviewer (ZW).

Certainty of evidence for each network comparison was assessed using the CINeMA framework across six domains (within-study bias, reporting bias, indirectness, imprecision, heterogeneity, and incoherence) (24, 25). Domain judgements were made according to CINeMA guidance and summarised into an overall certainty rating. Overall certainty was operationalised as: High (no downgrades), Moderate (downgraded in one domain), Low (downgraded in two domains), and Very Low (downgraded in three or more domains). Downgrading for within-study bias reflected the Cochrane risk-of-bias assessments. Imprecision was downgraded when the 95% confidence interval crossed the ±0.50 SMD range. Heterogeneity was downgraded when *I*^2^ exceeded 50%. Specific reasons for downgrading each comparison are summarized in Supplementary Tables S6–S7. We mapped our RoB assessments to the within-study bias domain at the study–treatment-comparison level; trials judged at high risk of bias or with some concerns were reflected accordingly in CINeMA, thereby influencing the overall confidence for each network estimate. Because pain intensity and disability were synthesized as standardized mean differences (SMDs) and no single anchor-based MCID applies consistently across the contributing scales, we used a distribution-based rule of thumb and pre-specified a range of equivalence (RoE) of ±0.50 SMD (26–28). For each network estimate, imprecision was judged according to the CINeMA framework as follows: no concerns when the 95% confidence interval (CI) lay entirely within the pre-specified range of equivalence (RoE; [−0.50, +0.50]) or entirely outside the RoE on one side (indicating a consistent clinically important effect); some concerns when the CI crossed one boundary of the RoE; and major concerns when the CI extended beyond both boundaries, making clinically important effects in opposite directions plausible (25). Any discrepancies in CINeMA judgments were resolved by discussion between two reviewers (HW and PD), with consensus determining the final overall confidence rating (high, moderate, low, or very low).

### 2.5 Statistical analyses

All statistical analyses were conducted using Stata version 18.0 (StataCorp, College Station, TX, United States) within the frequentist framework of network meta-analysis (NMA) (29). Because outcomes used different scales, we expressed effects as standardized mean differences (SMDs) computed as Hedges’ *g* and report 95% confidence intervals; positive values indicate improvement. A network diagram was constructed to visualize the geometry of the available evidence, where nodes represent interventions and edges indicate direct comparisons; the thickness of each edge was proportional to the number of trials for that comparison, and the size of each node reflected the total sample size.

A random-effects network meta-analysis model was applied, with between-study heterogeneity estimated as the between-study variance (*τ*^2^) using restricted maximum likelihood (REML) (30). Overall heterogeneity was quantified using the *I*^2^ statistic, calculated from the ratio of between-study variance to total variance, and interpreted as: 0–30% (low), 30–50% (moderate), 50–75% (substantial), and >75% (considerable) heterogeneity. We used a design-by-treatment interaction model (Wald test) and the Cochrane Q statistic to assess global inconsistency (defined as discords between direct and indirect evidence) and to examine local inconsistency using node-splitting and loop-specific methods (31). For the loop-specific approach, IFs and their 95% CIs were calculated, and loops with 95% CIs containing zero were deemed consistent. In the absence of significant inconsistency (all *p* > 0.05), all results are presented under the consistency model.

**Figure 1:**
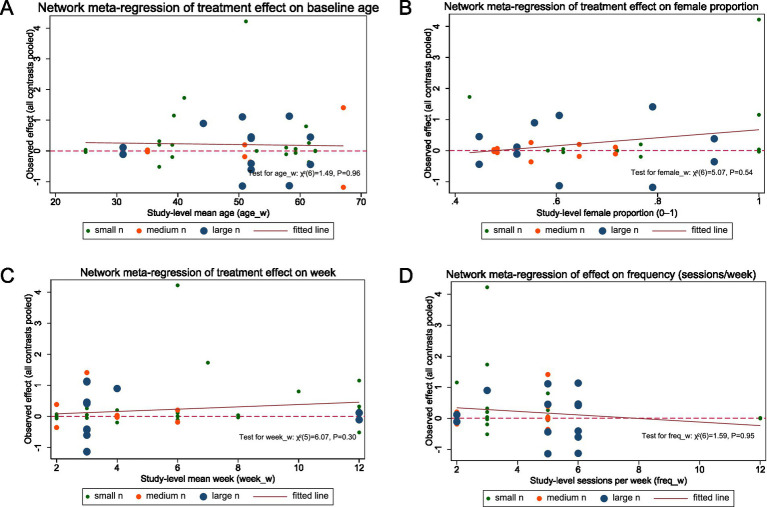
Network meta-regression for pain intensity reduction. Panels **(A–D)** plot the association between study-level covariates and treatment effects under a random-effects consistency NMA with a shared slope across comparisons: **(A)** baseline mean age (years), **(B)** female proportion (0–1), **(C)** intervention duration (weeks), and **(D)** training frequency (sessions/week). Each dot represents a study (points sized by total sample size: small/medium/large tertiles). The solid line is the fitted common-slope meta-regression; the dashed horizontal line marks the no-effect value (SMD = 0; positive values indicate benefit).

**Figure 2:**
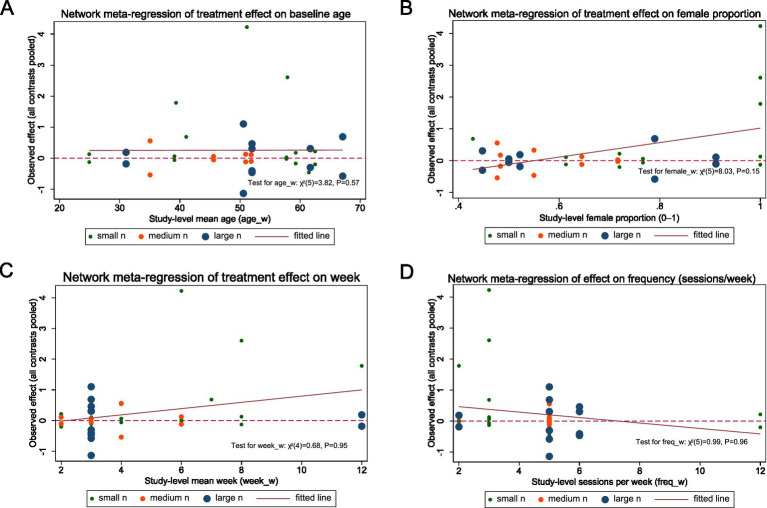
Network meta-regression for disability reduction. Panels **(A–D)** plot the association between study-level covariates and treatment effects under a random-effects consistency NMA with a shared slope across comparisons: **(A)** baseline mean age (years), **(B)** female proportion (0–1), **(C)** intervention duration (weeks), and **(D)** training frequency (sessions/week). Each dot represents a study (points sized by total sample size: small/medium/large tertiles). The solid line is the fitted common-slope meta-regression; the dashed horizontal line marks the no-effect value (SMD = 0; positive values indicate benefit).

We tested the validity of the transitivity assumption by assessing whether study-level covariates differed greatly across treatment comparisons (32). For each covariate, we performed meta-regression analyses with the treatment effect as the dependent variable and the covariate as the independent variable. The predefined study-level covariates were: mean age, gender, intervention week, and frequency of intervention. Full meta-regression results are reported in Figures 1, 2. For all trials, we analysed change-from-baseline (i.e., pre-to-post intervention variations) using data for these two comparators within each arm. When SDs of the pre-to-post intervention change were reported, we used them directly. When SDs of the change were not available, they were derived from baseline and post-treatment SDs using the standard Cochrane formula with an assumed pre–post correlation of *r* = 0.5: SD_change_ = √(SD_baseline_^2^ + SD_final_^2^–2 × *r*× SD_baseline_ × SD_final_). If a study reported arm-level standard errors (SEs) instead of SDs, we converted them using SD = SE × √n, where n is the number of participants in that arm. When the labelling of SE versus SD appeared inconsistent, we cross-checked the values against plausible ranges; studies for which no reasonable interpretation was possible were excluded from the quantitative synthesis. All pain and disability outcomes (VAS and NRS for pain; ODI and RMDQ for disability) were expressed as within-arm change (improvement) scores, defined so that higher values reflected greater clinical improvement (e.g., larger reductions in pain or disability). Treatment effects (Hedges’ *g*) were then calculated from these improvement scores, so that larger (more positive) SMD values indicate better outcomes in the experimental intervention compared with the comparator. All baseline and post-treatment scores within each trial were reported on the same scale, so no numerical unit conversions were required. The primary time point for all outcomes was predefined as the post-intervention assessment closest to the end of the treatment period. When multiple follow-up assessments were reported, only this immediate post-treatment time point was used in the main network meta-analysis. This time point was selected because it was consistently reported and methodologically comparable across the included studies, whereas longer-term follow-up assessments varied substantially in timing and were therefore not sufficiently homogeneous for inclusion. Besides, we conducted a sensitivity analysis for omissions, in which each study was sequentially excluded to assess its impact on the pooled estimate. This approach enabled us to identify studies that had a disproportionate impact on the overall robustness of the model. To aid clinical interpretation, we back-translated pooled SMDs to a 0–100 pain metric by multiplying the pooled SMD by a representative SD (median SD across included trials after rescaling 0–10 scales to 0–100) (33).

To summarize comparative performance across the whole network, we calculated three complementary ranking measures:

(i) Probability of being best (PrBest) – the chance that a treatment ranks first among all options;

(ii) Mean rank – the average position a treatment occupies across all possible rankings (lower is better);

(iii) Surface under the cumulative ranking curve (SUCRA) – a 0–1 summary that tells how close a treatment is to the best (1) versus the worst (0) rank. A higher SUCRA means a greater likelihood of being among the more effective options (e.g., SUCRA ≈ 0.80 indicates most of its ranking probability lies in the upper part of the hierarchy) (34).

We plotted cumulative ranking curves (curves further to the upper-left denote better ranks). Because ranks do not quantify effect size, they were interpreted alongside the pairwise SMDs and 95% CIs. Contribution plots illustrated how each direct comparison informed the network estimates. Potential publication bias was explored using comparison-adjusted funnel plots and Egger’s test. Statistical significance was set at *α* = 0.05 (two-sided).

## 3 Results

### 3.1 Search results

**Figure 3:**
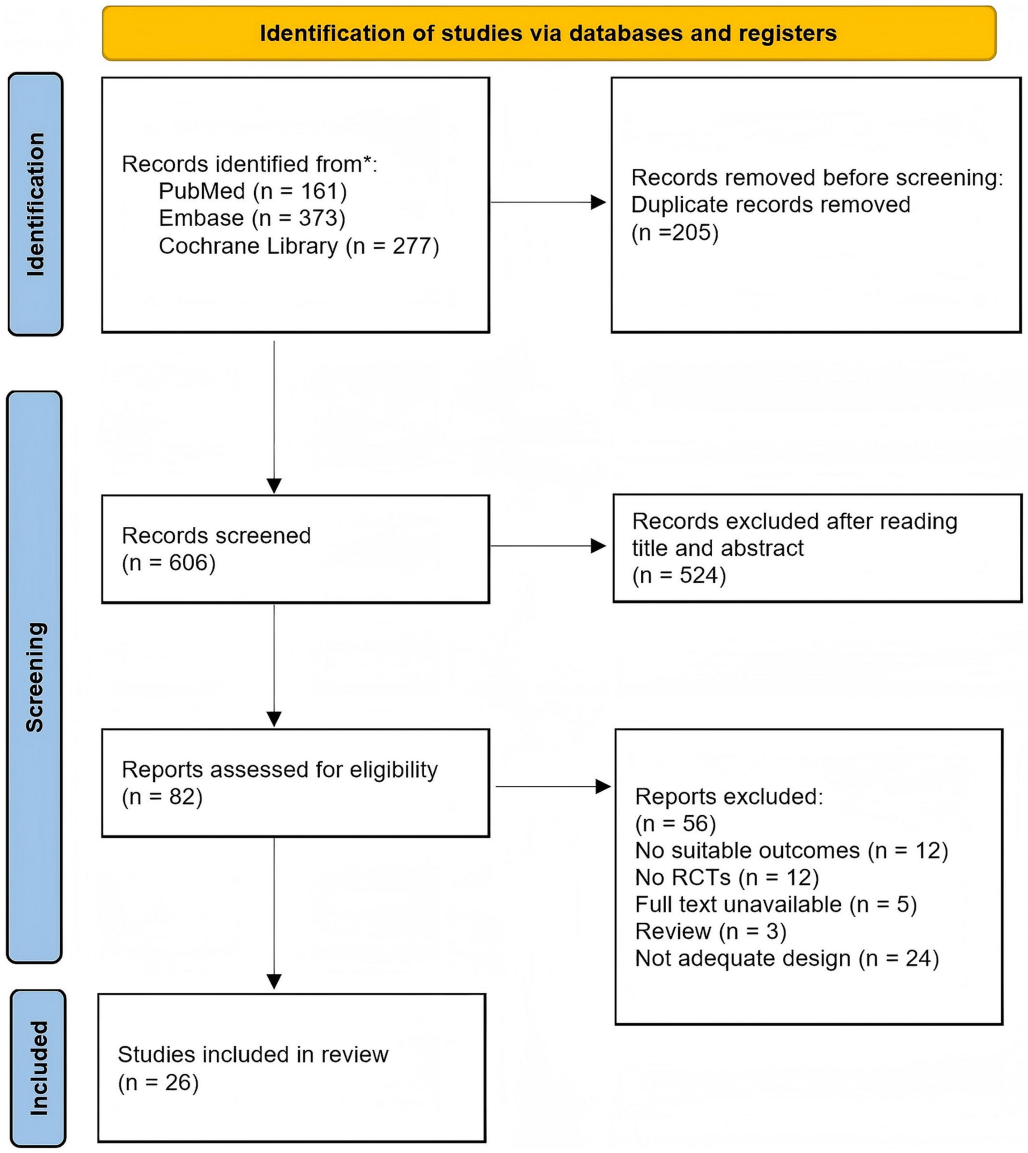
PRISMA flow diagram of study selection. The diagram illustrates the number of records identified, screened, assessed for eligibility, and included in the review, along with reasons for exclusion at each stage.

According to the pre-designed literature search strategy, 811 documents were retrieved, of which 205 were duplicates. After removing duplicates, 606 records were screened by title and abstract, and 524 were excluded. A total of 82 full-text articles were assessed for eligibility, of which 56 were excluded. Finally, 26 RCTs (11–17, 35–53) involving patients with CLBP were included in the review. The literature screening process and results are presented in Figure 3.

A total of nine interventions (network nodes) were prespecified and identified across the included trials: blank control, general care, land-based exercise, aquatic exercise, balneotherapy, balneotherapy + general care, balneotherapy + land-based exercise, land-based exercise + general care, and aquatic exercise + general care.

A total of 24 RCTs contributed 49 trial arms and 1,803 participants to the pain network (23 two-arm and 1 three-arm trials). The nine prespecified interventions (network nodes) were represented as follows: blank control (BC) 5 trials (*n* = 109); general care (GC) 9 trials (*n* = 537); land-based exercise (LE) 8 trials (*n* = 172); aquatic exercise (AE) 12 trials (*n* = 257); balneotherapy (BT) 2 trials (*n* = 59); BT + GC 7 trials (*n* = 497); BT + LE 4 trials (*n* = 112); LE + GC 1 trial (*n* = 30); and AE + GC 1 trial (*n* = 30).

A total of 21 RCTs contributed 42 trial arms and 1,612 participants (all two-arm trials) for disability network. Node-level representation was: BC 4 trials (*n* = 56); GC 7 trials (*n* = 465); LE 7 trials (*n* = 182); AE 11 trials (*n* = 268); BT 1 trial (*n* = 24); BT + GC 6 trials (*n* = 445); BT + LE 4 trials (*n* = 112); LE + GC 1 trial (*n* = 30); AE + GC 1 trial (*n* = 30).

Among the 26 included trials, 19 recruited both male and female participants (11–15, 37, 39, 40, 42–51, 53), 4 enrolled only female participant (16, 17, 35, 52), and 3 included not record of participants’ gender (36, 38, 41). Female participants accounted for 54.6% of the total sample. The mean of the minimum reported participant ages across studies was 23.1 years, while the mean of the maximum reported ages was 68.4 years. Programs typically involved supervised sessions two to 12 times per week, over periods ranging from 2 to 12 weeks. While protocols varied, all subjects were adults aged 24.5 to 67.4 years, using pain intensity and disability as primary outcomes. All included participants met the clinical definition of CLBP, characterized by a duration of ≥12 weeks and confirmed by general practitioner diagnosis (Table 1).

**Table 1:** Overview of included studies.

Study	Country	Sample size (female)	Age (years)	Duration of pain	Interventions	Intervention time	Outcome
Abadi 2019 (17)	Malaysia	39 (39) EG: 19 (19) CG: 20 (20)	EG: 37.85 ± 5.83 CG: 40.8 ± 5.25	24 weeks	EG: aquatic exercise CG: blank control	60 min, 2 times/week, 12 weeks	① ②
Alikhajeh 2020 (35)	Iran	24 (24) EG: 12 (12) CG: 12 (12)	EG: 51.6 ± 3.8 CG: 50.7 ± 3.3	12 weeks	EG: aquatic exercise CG: blank control	60 min, 3 times/week, 6 weeks	① ②
Ansari 2021 (16)	Iran	20 (20) EG: 10 (10) CG: 10 (10)	EG: 58.8 ± 3.73 CG: 57 ± 4.09	NR	EG: aquatic exercise CG: blank control	60 min, 3 times/week, 8 weeks	②
Bayattork 2022 (36)	Iran	60 (NR) EG1: 20 (NR) EG2: 20 (NR) EG3: 20 (NR)	EG1: 38.5 ± 12.68 EG2: 35.1 ± 15.12 EG3: 37.3 ± 13.11	12 weeks	EG1: land-based exercise EG2: aquatic exercise EG3: general care	20-60 min, 3 times/week, 12 weeks	①
Bello 2010 (37)	Ghana	12 (7) EG: 6 (4) CG: 6 (3)	EG: 53 ± 8.67 CG: 52.8 ± 12.37	12 weeks	EG: aquatic exercise CG: land-based exercise	60 min, 2 times/week, 6 weeks	①
Constant 1997 (53)	France	126 (94) EG: 63 (NR) CG: 63 (NR)	52	48 weeks	EG: balneotherapy + general care CG: general care	32.5 min, 6 times/week, 3 weeks	① ②
Constant 1998 (12)	France	219 (143) EG: 125 (NR) CG: 94 (NR)	52	48 weeks	EG: balneotherapy + general care CG: general care	45 min, 6 times/week, 3 weeks	① ②
Demirel 2008 (13)	Turkey	44 (27) EG: 23 (14) CG: 21 (13)	NR	12 weeks	EG: balneotherapy + land-based exercise CG: land-based exercise	25 min, 5 times/week, 3 weeks	① ②
Dilekci 2020 (38)	Turkey	262 (NR) EG: 133 (NR) CG: 129 (NR)	EG: 51.69 ± 10.26 CG: 49.42 ± 10.86	12 weeks	EG: balneotherapy + general care CG: general care	20 min, 5 times/week, 3 weeks	① ②
Dogan 2011 (39)	Turkey	60 (33) EG: 35 (21) CG: 25 (12)	EG: 61.5 ± 8.8 CG: 61.4 ± 8.8	48 weeks	EG: balneotherapy + general care CG: general care	20 min, 5 times/week, 3 weeks	① ②
Dundar 2009 (40)	Turkey	65 (31) EG: 32 (15) CG: 33 (16)	EG: 35.3 ± 7.8 CG: 34.8 ± 8.3	36 weeks	EG: aquatic exercise CG: land-based exercise	60 min, 5 times/week, 4 weeks	① ②
Gati 2018 (15)	Hungary	105 (47) EG: 52 (22) CG: 53 (25)	EG: 62.94 ± 9.3 CG: 60.49 ± 11.81	12 weeks	EG: balneotherapy + general care CG: general care	20 min, 5 times/week, 3 weeks	① ②
Guillemin 1994 (11)	France	104 (42) EG: 52 (34) CG: 52 (29)	EG: 58.8 ± 2.3 CG: 57.7 ± 2.3	96 weeks	EG: balneotherapy + general care CG: general care	20 min, 6 times/week, 3 weeks	①
Gunsoo 2011 (41)	Korea	19 (NR) EG: 9 (NR) CG: 10 (NR)	EG: 61.2 ± 3.3 CG: 60.8 ± 5.0	NR	EG: aquatic exercise CG: blank control	50 min, 5 times/week, 10 weeks	①
Kesiktas 2012 (42)	Turkey	60 (29) EG: 30 (16) CG: 30 (13)	EG: 58.43 ± 7.92 CG: 60.12 ± 9.83	8 weeks	EG: balneotherapy + land-based exercise CG: land-based exercise + general care	30 min, 5 times/week, 2 weeks	① ②
Konard 1992 (43)	Hungary	88 (49) EG: 35 (20) CG: 53 (29)	EG: 49 ± 8.8 CG: 41 ± 8.9	12 weeks	EG: balneotherapy CG: blank control	15 min, 3 times/week, 4 weeks	①
Mirmoezzi 2021 (44)	Iran	28 (12) EG: 14 (5) CG: 14 (7)	EG: 42.99 ± 4.55 CG: 39.12 ± 6.12	12 weeks	EG: aquatic exercise CG: blank control	60 min, 3 times/week, 7 weeks	① ②
Nemcic 2013 (45)	Croatia	72 (36) EG: 36 (18) CG: 36 (18)	EG: 41.61 ± 8.24 CG: 49.61 ± 7.98	12 weeks	EG: aquatic exercise CG: land-based exercise	45 min, 5 times/week, 3 weeks	②
Onat 2014 (46)	Turkey	81 (64) EG: 37 (27) CG: 44 (37)	EG: 67.43 ± 6.20 CG: 66.81 ± 9.77	32 weeks	EG: balneotherapy + general care CG: general care	20 min, 5 times/week, 3 weeks	① ②
Peng 2022 (47)	China	113 (59) EG: 56 (26) CG: 57 (33)	EG: 31.7 ± 11.3 CG: 30.4 ± 11.8	12 weeks	EG: aquatic exercise CG: general care	60 min, 2 times/week, 12 weeks	① ②
Pires 2015 (48)	Portugal	62 (40) EG: 30 (20) CG: 32 (20)	EG: 50.9 ± 6.2 CG: 51.0 ± 6.3	96 weeks	EG: aquatic exercise + general care CG: aquatic exercise	30-50 min, 2 times/week, 6 weeks	① ②
Sjogren 1997 (49)	Australia	60 (43) EG: 30 (21) CG: 30 (22)	EG: 58.11 ± 11.60 CG: 57.36 ± 13.59	24 weeks	EG: aquatic exercise CG: land-based exercise	50 min, 2 times/week, 6 weeks	① ②
Takinaci 2019 (50)	Turkey	50 (36) EG: 26 (18) CG: 24 (18)	EG: 64.5 ± 11.9 CG: 60.33 ± 9.6	24 weeks	EG: balneotherapy + land-based exercise CG: balneotherapy	20 min, 12 times/week, 2 weeks	① ②
Yalfani 2020 (52)	Iran	24 (24) EG: 12 (12) CG: 12 (12)	EG: 25.17 ± 3.04 CG: 24.67 ± 4.96	12 weeks	EG: aquatic exercise CG: land-based exercise	75 min, 3 times/week, 8 weeks	① ②
Yozbatiran 2004 (51)	Turkey	30 (23) EG: 15 (10) CG: 15 (13)	EG: 39.60 ± 6.33 CG: 38.60 ± 6.57	12 weeks	EG: aquatic exercise CG: land-based exercise	3 times/week, 4 weeks	① ②
Yucesoy 2021 (14)	Turkey	68 (62) EG: 33 (28) CG: 35 (34)	EG: 51.24 ± 11.26 CG: 52.46 ± 12.65	52 weeks	EG: balneotherapy + land-based exercise CG: land-based exercise	20 min, 5 times/week, 2 weeks	① ②

EG, experimental group; CG, control group; NR, not reported.

① Primary outcome: pain intensity.

② Secondary outcome: disability.

Intervention time is presented as session duration, frequency, and total intervention period.

Age is presented as mean ± standard deviation.

### 3.2 Risk of bias and certainty of evidence

**Figure 4:**
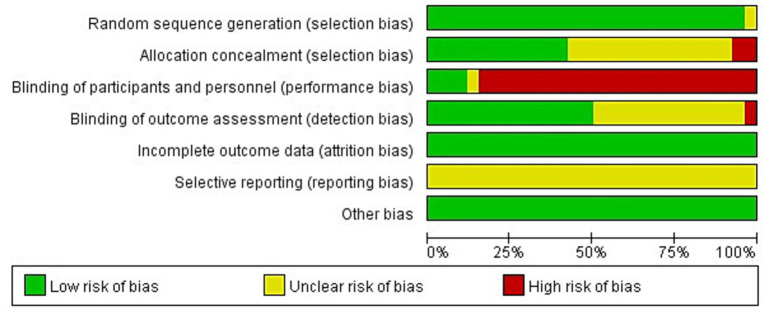
Risk of bias summary assessed using the Cochrane RoB tool for included randomized controlled trials.

**Figure 5:**
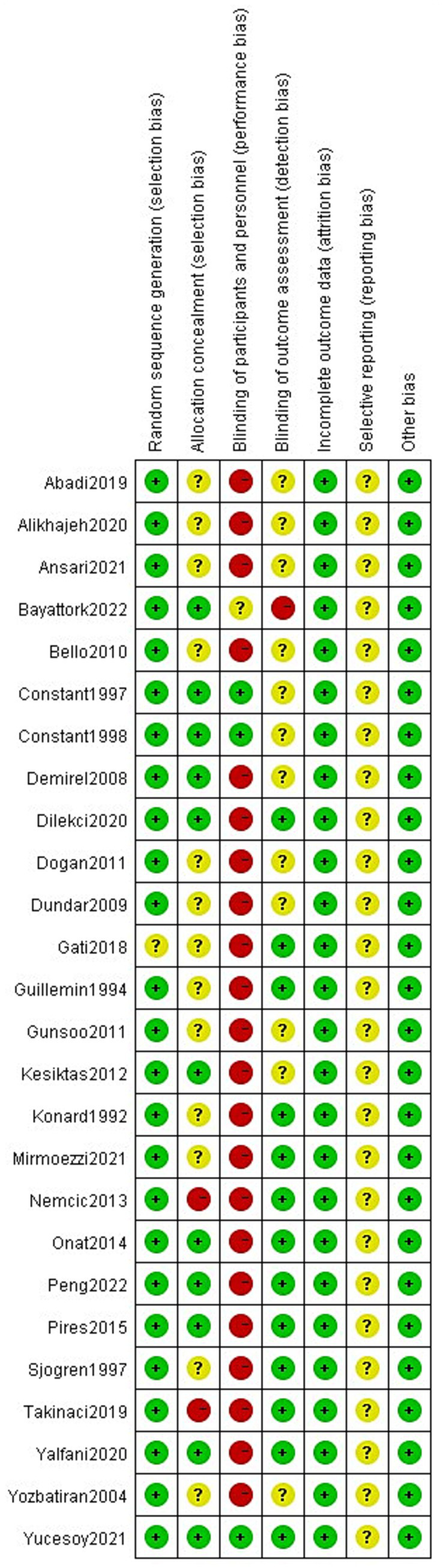
Study-level risk of bias judgments (Cochrane RoB tool).

Risk of bias was assessed using the Cochrane RoB tool across standard domains (random sequence generation, allocation concealment, blinding of participants/personnel, blinding of outcome assessment, incomplete outcome data, selective reporting, and other bias; Figures 4, 5). Overall, many trials reported an adequate randomization approach, whereas allocation concealment was frequently insufficiently described. Blinding of participants and personnel was commonly judged at high risk, reflecting the practical difficulty of blinding in exercise- and spa-based interventions. For subjective outcomes such as pain and disability, blinding of outcome assessment was often unclear or not feasible, depending on trial procedures and reporting. Attrition bias was generally low, while selective reporting was frequently judged as unclear due to limited protocol/registration information. Other sources of bias were uncommon. Detection bias risk may systematically differ between aquatic and land-based interventions. Aquatic therapies’ distinctive sensory characteristics (warmth, immersion, odor) may make concealment from outcome assessors more difficult than land-based exercise, potentially inflating perceived effectiveness for water-based modalities. However, in this dataset, high-risk ratings for outcome-assessor blinding were similarly common in aquatic and land-based trials (84.6% vs. 81.8%, respectively).

**Figure 6:**
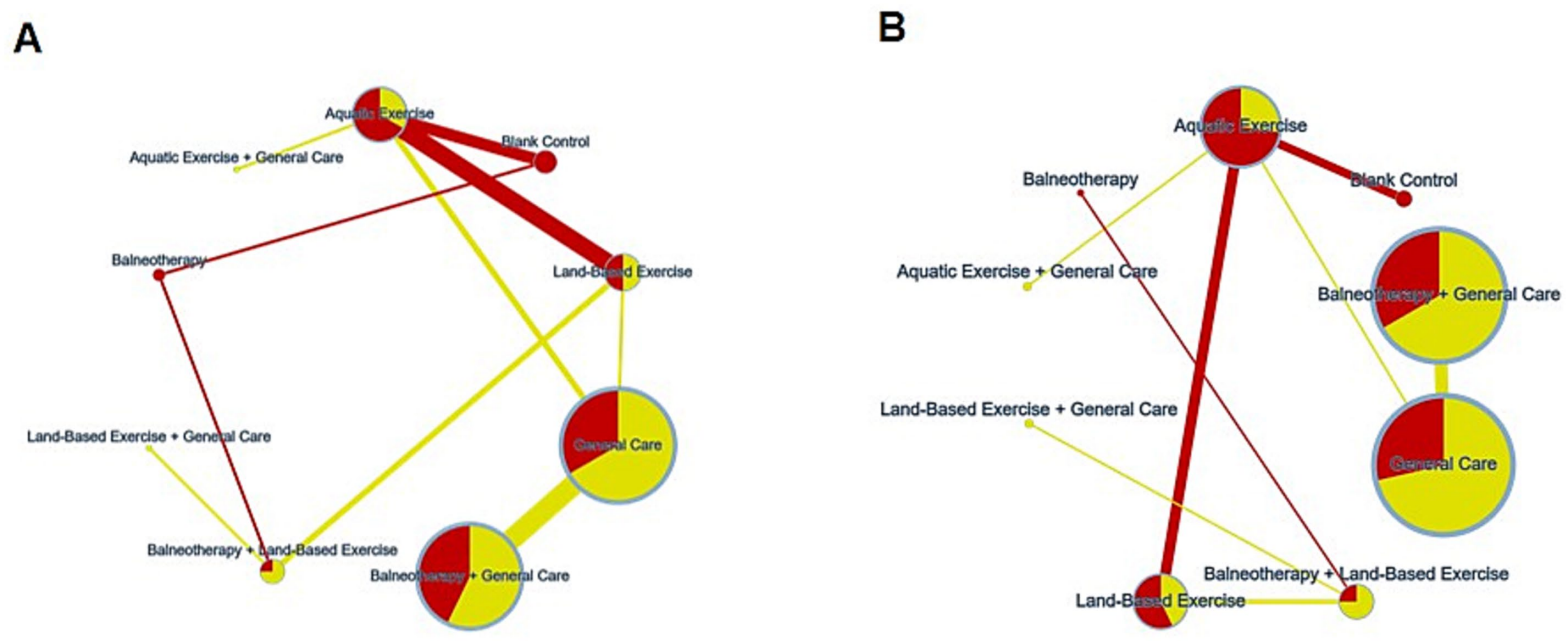
Distribution of risk of bias within the networks (yellow, low risk; red, some concerns/high risk; edge color reflects the overall risk of bias of the contributing studies). **(A)** pain reduction; **(B)** disability reduction.

For both pain intensity and disability, the certainty ranged from moderate to very low. For pain intensity, downgrading was driven mainly by within-study bias (RoB) and by imprecision judged against the pre-specified equivalence range (±0.50 SMD); downgrading for heterogeneity occurred occasionally, whereas incoherence signals were limited and mostly seen in sparsely informed comparisons. For disability, certainty was also limited predominantly by imprecision (wide intervals around small effects) and, to a lesser extent, within-study bias; incoherence and indirectness contributed minimally (Figure 6; Supplementary Tables S6, S7).

### 3.3 Network geometry

**Figure 7:**
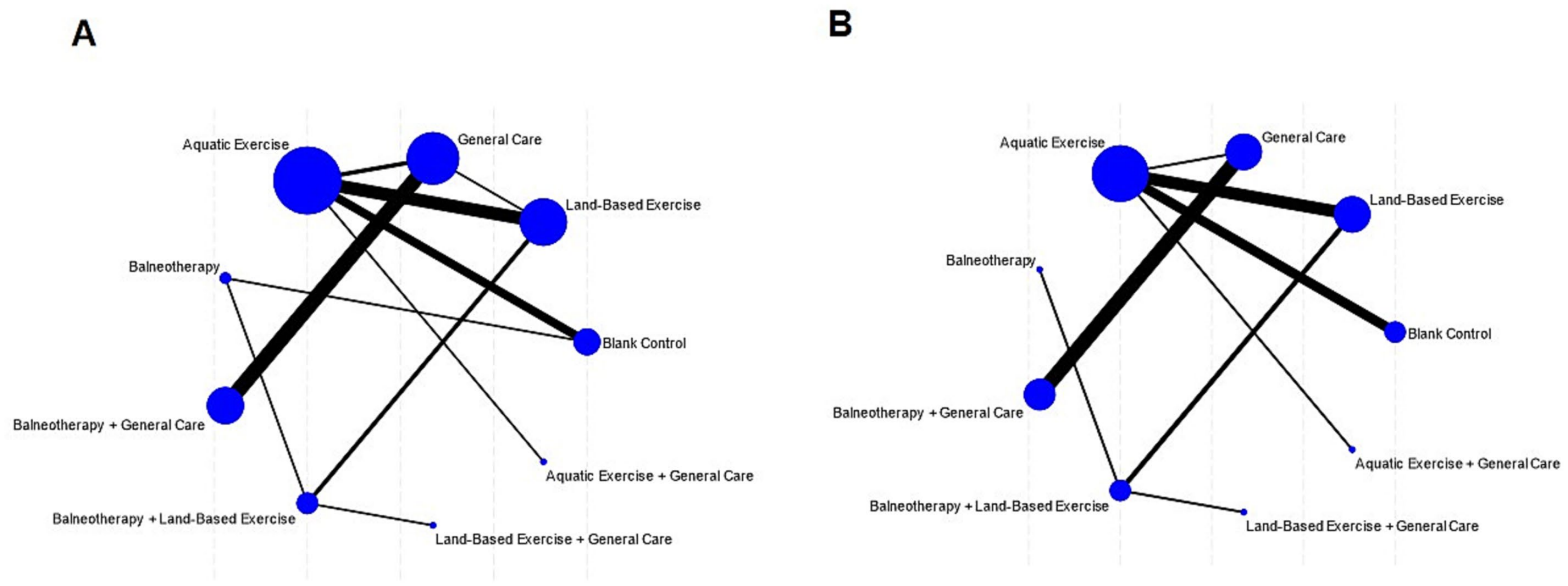
Network geometry of randomized trials in adults with chronic non-specific low back pain (CLBP). **(A)** Pain intensity; **(B)** disability. Nodes denote interventions (abbreviations/labels as defined in Section 2.2.1). Node size is proportional to the total number of participants randomized to that node across contributing trials; edge thickness is proportional to the number of head-to-head trials informing the comparison.

Two independent networks were constructed for the outcomes of pain intensity reduction and disability reduction (Figure 7). Each node represents a unique intervention, and each edge indicates a direct comparison between interventions. Node size is proportional to the total sample size for that intervention, and edge thickness corresponds to the number of trials comparing the connected interventions.

The networks for pain intensity reduction and disability reduction both included nine interventions and were well connected, enabling both direct and indirect comparisons with the blank control group as the reference comparator.

### 3.4 Network meta-analysis

#### 3.4.1 Pain intensity reduction

**Figure 8:**
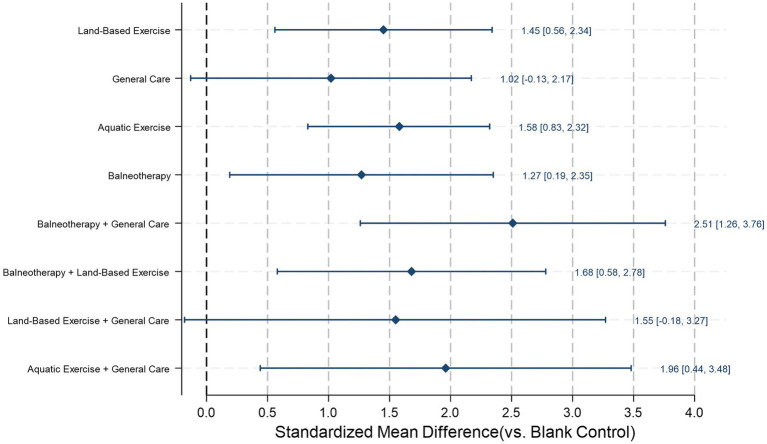
Pain reduction in adults with chronic non-specific low back pain (CLBP) at post-intervention: network estimates versus blank control. Diamonds show standardized mean differences (SMDs) with 95% CIs from a frequentist random-effects network meta-analysis; positive SMD indicates less pain. The vertical dashed line (0) denotes no difference versus the references (blank control).

We pooled data from 24 studies and found that, compared with the blank control group, balneotherapy + general care (SMD = 2.51, 95% CI 1.26–3.76; *p* < 0.001), aquatic exercise + general care (SMD = 1.96, 95% CI 0.44–3.48; *p* = 0.011), balneotherapy + land-based exercise (SMD = 1.68, 95% CI 0.58–2.78; *p* = 0.003), aquatic exercise (SMD = 1.58, 95% CI 0.83–2.32; *p* < 0.001), land-based exercise (SMD = 1.45, 95% CI 0.56–2.34; *p* = 0.001), and balneotherapy (SMD = 1.27, 95% CI 0.19–2.35; *p* = 0.021) were associated with significant reductions in pain intensity. By contrast, land-based exercise + general care (SMD = 1.55, 95% CI − 0.18–3.27; *p* = 0.078) and general care (SMD = 1.02, 95% CI − 0.13–2.17; *p* = 0.081) may also reduce pain, but did not reach statistical significance (Figure 8). Land-based exercise + general care apparent underperformance is based on sparse evidence (one small trial; *n* = 60, 30 per group) and variability in what constituted ‘general care’ across trials. In the contributing trial, the comparator program comprised a short, 2-week course (5 sessions/week) of physiotherapy modalities (TENS, ultrasound, infrared) alongside an exercise program, which may have limited the opportunity for additive effects.

For clinical context, using a representative SD of 19.2 points on a 0–100 pain scale, the observed SMDs correspond approximately to 24–48 points of pain reduction across the interventions that showed statistically significant effects (e.g., balneotherapy + general care: SMD = 2.51 = 48.19 points; aquatic exercise: SMD = 1.58 = 30.33 points; balneotherapy: SMD = 1.27 = 24.38). Land-based exercise + general care yielded a similar point estimate (SMD = 1.55 ≈ 29.76 points) but did not reach statistical significance because it was informed by sparse evidence (one small trial) and was accompanied by wide confidence intervals that crossed the null—a distinction critical for interpretation.

**Figure 9:**
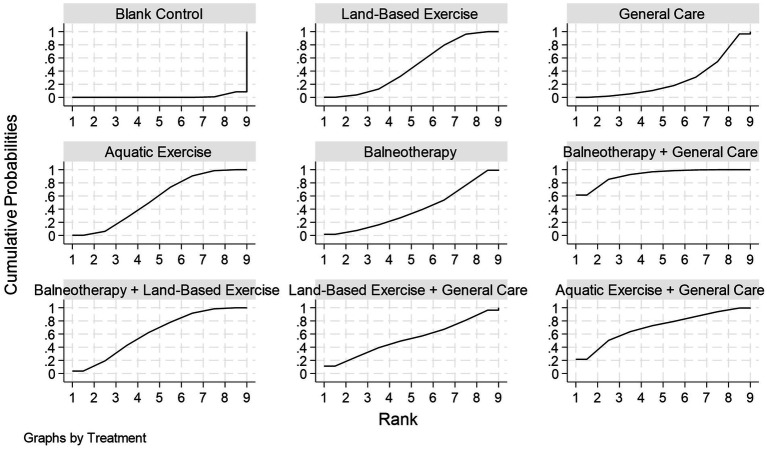
Cumulative ranking curves (rankograms) for pain intensity in adults with chronic non-specific low back pain (CLBP). Panels show each intervention’s cumulative probabilities. The *x*-axis shows possible ranks; the *y*-axis shows the cumulative probability of achieving a given rank or better. Curves closer to the upper-left indicate a higher probability of top ranks and therefore a larger SUCRA value (0–1). Corresponding SUCRA, PrBest, and mean rank are reported in Table 2. Estimates come from a frequentist random-effects network meta-analysis.

Based on SUCRA rankings (Table 2; Figure 9), balneotherapy + general care performed best (SUCRA = 0.92; PrBest = 61.4%; mean rank = 1.7). It was followed by aquatic exercise + general care (0.71; 21.5%; 3.3), balneotherapy + land-based exercise (0.62; 3.8%; 4.0), and land-based exercise + general care (0.53; 11.2%; 4.7), indicating that combined interventions generally ranked above single modalities. Among single interventions, aquatic exercise (0.56; 0.2%; 4.5) and land-based exercise (0.48; 0.2%; 5.2) ranked higher than balneotherapy (0.40; 1.7%; 5.8) and general care (0.27; 0.0%; 6.8). Blank control (0.01; 0.0%; 8.9) consistently ranked last, supporting its minimal effectiveness for pain reduction.

**Table 2:** SUCRA, probability of best, and mean rank for pain reduction interventions.

Intervention	Number of studies	SUCRA (0–1)	PrBest (%)	Mean rank
Blank control	5	0.01	0.0	8.9
Land-based exercise	8	0.48	0.2	5.2
General care	9	0.27	0.0	6.8
Aquatic exercise	12	0.56	0.2	4.5
Balneotherapy	2	0.4	1.7	5.8
Balneotherapy + general care	7	0.92	61.4	1.7
Balneotherapy + land-based exercise	4	0.62	3.8	4.0
Land-based exercise + general care	1	0.53	11.2	4.7
Aquatic exercise + general care	1	0.71	21.5	3.3

SUCRA (surface under the cumulative ranking curve; range 0–1), higher values indicate a better overall ranking. PrBest (%), the probability that an intervention ranks first in the network. Mean rank, average position in the treatment hierarchy (1 = best). “+” indicates combined interventions. Interventions based on ≤3 trials should be interpreted with substantial caution, as exclusion of single trials would meaningfully alter rankings.

In the network meta-analysis for pain intensity reduction, substantial heterogeneity was observed (*τ*^2^ = 0.42; *I*^2^_res = 85.61%). Global inconsistency, assessed using the design-by-treatment interaction model, was not statistically significant (Wald *χ*^2^(3) = 1.34, *p* = 0.72). Local inconsistency was further evaluated via node-splitting and loop-specific approaches, with all inconsistency factors (IFs) having 95% CIs including zero, suggesting no evidence of significant local inconsistency.

#### 3.4.2 Disability reduction

**Figure 10:**
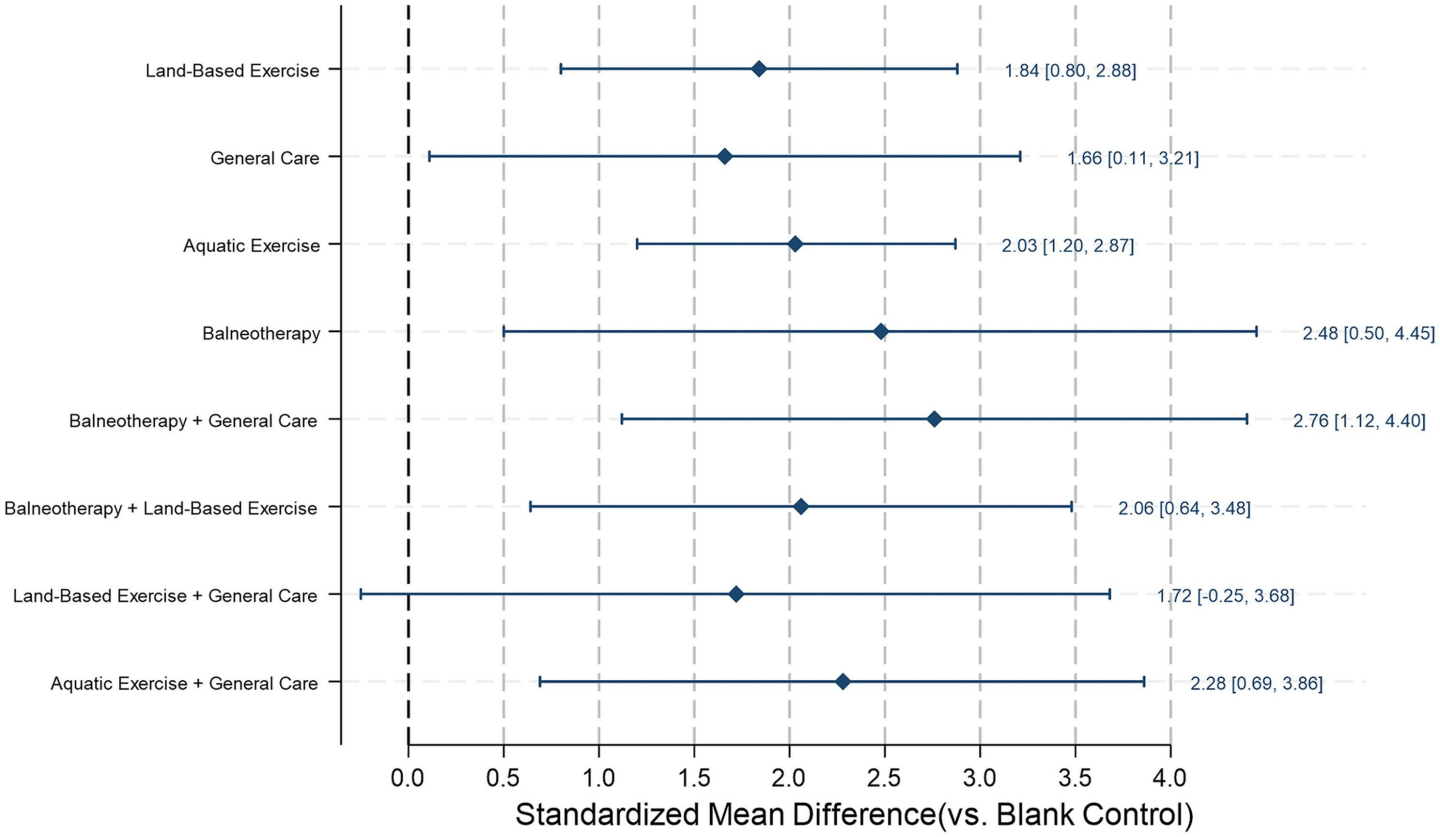
Disability reduction in adults with chronic non-specific low back pain (CLBP) at post-intervention: network estimates versus blank control. Diamonds show standardized mean differences (SMDs) with 95% CIs from a frequentist random-effects network meta-analysis; positive SMD indicates less pain. The vertical dashed line (0) denotes no difference versus the references (blank control).

We pooled data from 21 studies, which showed that compared with the blank control group, balneotherapy + general care (SMD = 2.76, 95% CI 1.12 to 4.40, *p* = 0.001), balneotherapy (SMD = 2.48, 95% CI 0.50 to 4.45, *p* = 0.014), aquatic exercise + general care (SMD = 2.28, 95% CI 0.69 to 3.86, *p* = 0.005), balneotherapy + land-based exercise (SMD = 2.06, 95% CI 0.64 to 3.48, *p* = 0.004), aquatic exercise (SMD = 2.03, 95% CI 1.20 to 2.87, *p* < 0.001), land-based exercise (SMD = 1.84, 95% CI 0.80 to 2.88, *p* = 0.001), and general care (SMD = 1.66, 95% CI 0.11 to 3.21, *p* = 0.035) were all associated with significant disability reduction. Land-based exercise + general care (SMD = 1.72, 95% CI − 0.25 to 3.68, *p* = 0.086) did not reach statistical significance (Figure 10).

**Figure 11:**
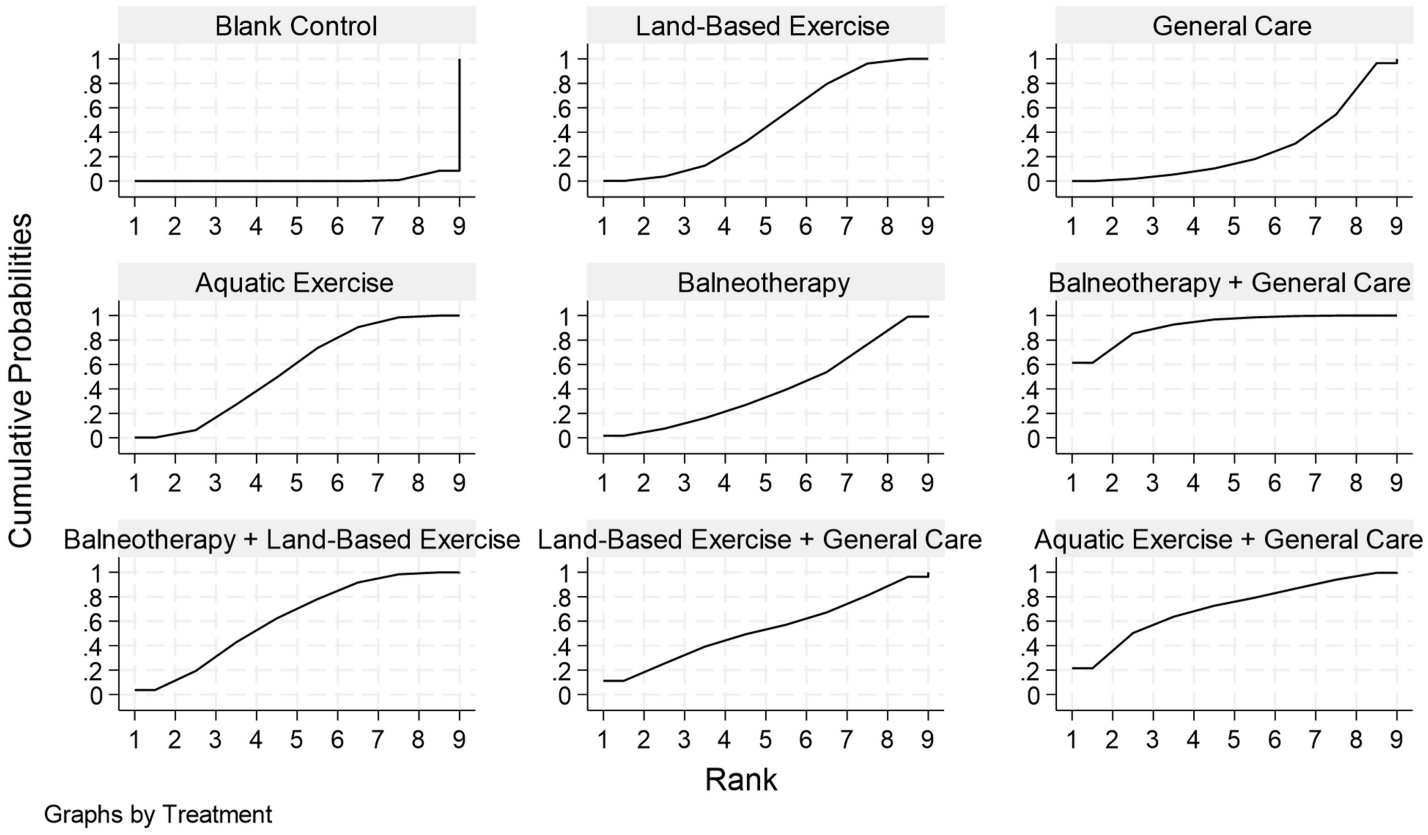
Cumulative ranking curves for each intervention in disability reduction. The *x*-axis represents the possible ranks of the interventions, and the *y*-axis represents the cumulative probability of achieving a given rank or better. A curve shifted toward the upper left corner indicates a greater likelihood of higher ranking, which corresponds to a higher surface under the cumulative ranking curve (SUCRA) value. Corresponding SUCRA, PrBest, and mean rank are reported in Table 3. Estimates come from a frequentist random-effects network meta-analysis.

Based on SUCRA (Table 3; Figure 11), balneotherapy + general care ranked first (SUCRA = 0.83; PrBest = 44.6%; mean rank = 2.4). It was followed by balneotherapy (0.71; 29.3%; 3.3) and aquatic exercise + general care (0.64; 16.9%; 3.9). Aquatic exercise (0.55; 1.1%; 4.6) and balneotherapy + land-based exercise showing moderate performance (0.55; 2.5%; 4.6). Lower ranks were observed for land-based exercise (0.41; 5.2%; 5.7), land-based exercise + general care (0.41; 5.2%; 5.7), and general care (0.38; 0.0%; 6.0). Blank control consistently ranked last (0.01; 0.0%; 8.9).

**Table 3:** SUCRA, probability of best, and mean rank for disability reduction interventions.

Intervention	Number of studies	SUCRA (0–1)	PrBest (%)	Mean rank
Blank control	4	0.01	0.0	8.9
Land-based exercise	7	0.42	0.4	5.6
General care	7	0.38	0.0	6.0
Aquatic exercise	11	0.55	1.1	4.6
Balneotherapy	1	0.71	29.3	3.3
Balneotherapy + general care	6	0.83	44.6	2.4
Balneotherapy + land-based exercise	4	0.55	2.5	4.6
Land-based exercise + general care	1	0.41	5.2	5.7
Aquatic exercise + general care	1	0.64	16.9	3.9

SUCRA (surface under the cumulative ranking curve; range 0–1), higher values indicate a better overall ranking. PrBest (%), the probability that an intervention ranks first in the network. Mean rank, average position in the treatment hierarchy (1 = best). “+” indicates combined interventions. Interventions based on ≤3 trials should be interpreted with substantial caution, as exclusion of single trials would meaningfully alter rankings.

In the network meta-analysis for disability reduction, substantial heterogeneity was observed (*τ*^2^ = 0.41; *I*^2^_res = 87.20%). Global inconsistency, assessed using the design-by-treatment interaction model, was not statistically significant (Wald *χ*^2^(1) = 0.25, *p* = 0.62). Similarly, local inconsistency was further evaluated via node-splitting, with all inconsistency factors (IFs) having 95% CIs including zero, suggesting no evidence of significant local inconsistency.

### 3.5 Publication bias, meta-regression and sensitivity analyses

**Figure 12:**
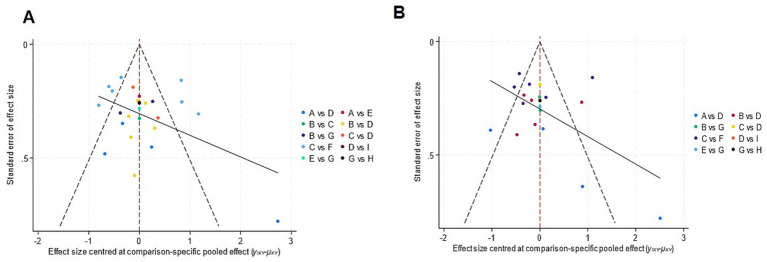
Funnel plots for pain intensity reduction **(A)** and disability reduction **(B)**. Each colored point represents a pairwise comparison between two interventions, with letters (A–I) denoting specific intervention groups. The vertical dashed red line represents the pooled effect size, the diagonal dashed lines indicate pseudo 95% confidence limits, and the solid black line represents the regression line for funnel plot asymmetry.

Funnel plots for pain intensity reduction, and disability reduction are presented in Figure 12. Visual inspection of the plots for pain intensity reduction and disability reduction revealed a generally symmetrical distribution of effect sizes around the pooled estimate. This impression was supported by Egger’s regression tests (*p* > 0.05 for both outcomes), suggesting a low likelihood of small-study effects or substantial publication bias.

None of the pre-specified study-level covariates (mean age, % female, intervention duration in weeks, training frequency in sessions/week) were associated with treatment effects in the random-effects NMA with a shared slope across comparisons in pain reduction. The fitted slopes were near zero and the 95% CIs had no effect (all *p* > 0.05). Joint Wald tests were non-significant for all covariates in the analysis of pain reduction: age *χ*^2^(6) = 1.49, *p* = 0.96; % female *χ*^2^(6) = 5.07, *p* = 0.54; duration (weeks) *χ*^2^(6) = 1.59, *p* = 0.95; frequency (sessions/week) *χ*^2^(5) = 6.07, *p* = 0.30 (Figures 1A–D).

Findings were consistent for disability reduction: no covariate showed evidence of association with treatment effects (all *p* > 0.05). Joint Wald tests for disability scores were: age *χ*^2^(5) = 3.82, *p* = 0.57; % female *χ*^2^(5) = 8.03, *p* = 0.15; duration (weeks) *χ*^2^(5) = 0.99, *p* = 0.96; frequency (sessions/week) *χ*^2^(4) = 0.68, *p* = 0.95 (Figures 2A–D).

Finally, we performed leave-one-out sensitivity analyses. Across deletions, the direction of effect was consistent and point estimates varied only modestly. For a few contrasts whose baseline 95% CIs were close to the null, some deletions produced marginal “crossing-the-null” behavior; in another few, uncertainty widened because network connectivity was reduced. None of these changes altered the qualitative conclusions, indicating the results are not driven by any single study (Supplementary Figures S1–S4).

## 4 Discussion

To our knowledge, this is the first network meta-analysis to simultaneously compare hydrotherapeutic, aquatic and land-based interventions (and their combinations with general care) for chronic non-specific low back pain across pain and disability outcomes within a single, connected network. Prior reviews were limited to pairwise comparisons or to single modalities, leaving a gap in relative effectiveness across the full range of options and in probabilistic ranking to guide clinical selection. Our study addresses this gap by integrating direct and indirect evidence and reporting both effect sizes and SUCRA-based rankings for practical decision-making. This network meta-analysis found that, for both pain intensity reduction and disability reduction in individuals with CLBP, combined interventions—particularly balneotherapy + general care, aquatic exercise + general care, and balneotherapy + land-based exercise—tended to rank highest in effectiveness. Among single-component therapies, aquatic exercise and balneotherapy generally achieved higher rankings than land-based exercise and general care, although the differences between these single interventions were small and may not be clinically meaningful. Across outcomes, general care was consistently among the lowest-ranked active treatments, and blank control groups performed worst overall, reinforcing the limited benefit of no active intervention. While pain and disability reduction rankings were broadly similar, disability outcomes placed both balneotherapy and aquatic exercise alone slightly higher, suggesting potential added value for functional recovery beyond pain relief. Besides, we found no evidence that the pre-specified transitivity modifiers (age, sex, duration, frequency) altered relative treatment effects for either pain or disability. Meta-regression revealed no significant associations between participant age, gender, intervention duration, or training frequency and pain reduction (all *p* > 0.05). This null finding indicates that measured trial characteristics do not substantially explain heterogeneity; unmeasured factors (clinician expertise, treatment fidelity, participant illness beliefs) likely drive residual variation, reinforcing that precision effect estimates cannot be derived from this dataset. Results were unchanged after joint testing and leave-one-out sensitivity analyses, and the model-predicted differences across the observed covariate ranges were small.

### 4.1 Overall treatment effects across outcomes

Across both pain and disability reduction outcomes, combined interventions integrating hydrotherapy with structured exercise or general care generally achieved the highest rankings. For pain reduction, balneotherapy + general care, aquatic exercise + general care, and balneotherapy + land-based exercise generally ranked among the top positions, while for disability, balneotherapy + general care remained highest, with balneotherapy and aquatic exercise + general care also showing relatively large improvements. These findings align with previous evidence in musculoskeletal and rheumatic conditions showing that multi-modal approaches, particularly those incorporating aquatic or thermal elements, can enhance treatment effects compared with single-modality programs (54, 55). Consistently, Tognolo et al. (56) have emphasized that a comprehensive approach integrating traditional balneotherapy modalities with rehabilitation interventions within the spa environment may effectively address rheumatic diseases and related disability by exploiting the synergies between thermal properties and exercise or physical therapy (57). Disability outcomes ranked aquatic interventions relatively higher than pain outcomes, suggesting water immersion may facilitate functional improvement through mechanisms distinct from pain relief. Clinically, this pattern indicates practitioners prioritizing functional restoration should preferentially select aquatic modalities, whereas for pain-dominant presentations, modality selection may be less critical.

A potential contributor to the superiority of such multi-modal programs is the unique physiological environment created by water immersion, which facilitates exercise performance and amplifies therapeutic effects. The potential mechanisms underlying the observed benefits are multifactorial. Hydrotherapy and aquatic exercise provide buoyancy, which reduces mechanical load on the spine and facilitates pain-free movement (58, 59). Warm water immersion may induce vasodilation and enhance local circulation (60), and the effects of hydrostatic pressure may help reduce swelling, provide isometric support to joints, and improve proprioception (61–63). Immersion in water naturally slows down movement patterns, allowing for more controlled assessment of movement effects, correction of compensatory mechanisms, and relearning of correct movement patterns (64). In addition to the biomechanical factors, there may be neurophysiological factors that may contribute to the effects of aquatic exercise. Aerobic activity increases serotonergic neurotransmission and receptor expression and modulates central pain perception (65). The combination of effects of buoyancy, warmth, and hydrostatic pressure may stimulate cutaneous thermo- and mechanoreceptors, which may in turn inhibit the transmission of nociceptor and peripheral pain signaling (66). This input may then mediate the release of endogenous opioid peptides, which may also help reduce the pain (67).

In addition to these mechanistic scores, the outcome rankings confirm that, aside from multimodal programs that include aquatic/thermal components, which generally have better outcomes than other programs, balneotherapy and aquatic exercise alone also have relatively high disability outcome rankings. This contrasts with land-based exercise + general care, which showed high effect sizes but only achieved marginal statistical significance. This discrepancy is more likely to reflect methodological and contextual factors than a genuine absence of therapeutic benefit. First, only a small number of trials assessed this combination, resulting in wide confidence intervals and limited statistical power.

Second, the content of “general care” varied widely across trials, which ranging from brief advice and reassurance to structured patient education and activity guidance. This variability also reflects a broader reality in clinical practice, where limited coordination and communication among health care professionals often lead to fragmented, monodisciplinary care with restricted time and resources (68). Such heterogeneity is likely to have diluted any incremental benefit of adding general care to land-based exercise programs. Thirdly, studies show that higher training frequency, training volume, or longer intervention duration are significantly associated with larger treatment effects in CLBP exercise trials (69, 70); inconsistent or low-dose delivery of the general care component could therefore attenuate the combined effect. Finally, it is possible that adding general care to a land-based program may yield smaller additive gains than adding it to aquatic-based interventions, as the latter provide unique mechanical unloading, hydrostatic support, and thermal properties that may accelerate symptom relief and functional gains (64, 71, 72). Nonetheless, given the scarcity of direct head-to-head evidence and heterogeneity in intervention protocols, these interpretations should be considered tentative and warrant confirmation in future well-powered RCTs. Although differences between single interventions such as aquatic exercise, balneotherapy, and land-based exercise were small and unlikely to be clinically meaningful, their ranking trends suggest that aquatic- and spa-based programs may confer additional benefits in certain patient populations. Future trials should investigate whether these advantages persist in the long term, and whether they are moderated by baseline disability level, psychological factors, or adherence to therapy protocols.

Our rankings suggest that multimodal programs—particularly balneotherapy combined with general care—tend to perform best across outcomes, with aquatic-based strategies also favorable. Although balneotherapy + general care ranked highest (pain SUCRA = 0.92; disability SUCRA = 0.83), the evidence base differed markedly across combination nodes (e.g., balneotherapy + general care: *N* = 7 trials for pain and *N* = 6 for disability, whereas aquatic exercise + general care and land-based exercise + general care were each informed by single trials). In a descriptive check comparing each combination node with its constituent single modalities, combinations showed higher SUCRA values in 12 of 16 comparisons (75%) across outcomes (pain: 7/8; disability: 5/8), suggesting a consistent directional advantage of multimodal care; however, individual combination rankings—particularly those supported by single trials—should be interpreted with caution because exclusion of one trial could meaningfully alter rankings. These findings support the use of integrated programs where feasible, while acknowledging that SUCRA reflects relative ordering under the network rather than absolute benefit. Choice of modality should consider patient preferences, access and cost, co-morbidities, and the precision of estimates in each contrast. Where balneotherapy is unavailable, aquatic or land-based exercise remain evidence-supported options; the added value of combining land-based exercise with general care in our network was imprecisely estimated, and any apparent differences should be interpreted cautiously.

### 4.2 Advantages and limitations

We assessed transitivity by examining available study-level characteristics (age, gender, intervention duration, and training frequency) across comparisons. Meta-regression did not identify significant associations between these covariates and treatment effects, and we did not observe marked imbalances in reported characteristics across comparisons. Nevertheless, because our search strategy targeted aquatic interventions, evidence informing non-aquatic nodes (land-based programs and general care) was restricted to trials that also included an aquatic arm, which may limit generalizability and could influence the validity of indirect comparisons involving non-aquatic nodes.

This analysis is substantially limited by very high heterogeneity (*I*^2^ > 85%) and predominantly very low certainty evidence. Of the 36 comparisons assessed for pain intensity, 94.4% (34/36) were rated low to very low certainty; for disability, all 36 comparisons were rated very low certainty. These evidence quality profiles severely restrict ranking reliability and recommendation strength. In addition, the certainty for several contrasts was constrained mainly by imprecision and, to a lesser extent, within-study bias (RoB), reflecting sparse evidence—particularly for land-based exercise + general care—yielding wide intervals around small effects. Besides, the content and intensity of “general care” varied across trials, and some land-based arms were home-based with limited reporting; this introduces classification uncertainty between general care and land-based exercise, and the potential impact on contrasts is uncertain. Despite random-effects modeling, notable residual heterogeneity remained for both pain and disability, plausibly due to differences in participants, intervention implementation (dose/frequency, supervision), and study quality.

We could not examine whether water characteristics (including mineral content) modify the effect of aquatic exercise, because such characteristics were seldom reported in adequate detail and the available trials did not permit a robust, comparable mineral- versus tap-water analysis. Moreover, there was substantial clinical heterogeneity within both the balneotherapy and aquatic exercise nodes. Balneotherapy programs differed in water composition (e.g., mineral content, temperature), delivery setting (individual bathtubs vs. pools), session duration, weekly frequency and total number of sessions, and aquatic exercise programs varied in the type, intensity and progression of water-based exercises. This heterogeneity represents a limitation of the present study, as subgroup analyses based on these characteristics were not feasible due to insufficient statistical power. Future studies should therefore aim to investigate more homogeneous interventions or specifically examine how individual characteristics of these water-based therapies influence clinical outcomes. Although random-effects models and exploratory meta-regression were applied, these variations are likely to contribute to residual heterogeneity and limit the extent to which pooled estimates and treatment rankings can be directly mapped onto any single, specific protocol.

10 of 26 trials (38.5%) reported follow-up beyond post-treatment; timing varied from 1–12 months, precluding durability synthesis. All conclusions reflect immediate post-treatment effects only. Whether benefits persist at 3, 6, or 12 months—essential for clinical sustainability assessment—remains unknown. Although some trials reported outcomes at later follow-up (from a few weeks up to 12 months), these data were available in relatively few studies, at heterogeneous time points and often with incomplete reporting. This precluded a robust and connected network for medium- or long-term outcomes. As a result, the durability of the observed benefits beyond the end of treatment remains uncertain and should be addressed in future trials and syntheses.

Finally, our electronic search targeted water-based therapies and we retained all randomized arms; therefore evidence for non-aquatic nodes (land-based, combined programs, general care) derives from trials that also included an aquatic arm, which may limit generalizability to settings without aquatic components. As a consequence, the evidence informing the land-based exercise and general care nodes is limited to those trials that also chose to evaluate an aquatic intervention and does not encompass the entire body of land-based exercise or general care for CLBP. Leave-one-out analyses suggested that no single study unduly influenced results, but they do not eliminate the above uncertainties.

A key strength of this study is its comprehensive scope, incorporating a wide range of hydrotherapeutic and exercise-based interventions relevant to real-world practice. By evaluating pain, and disability simultaneously, the analysis provides a multidimensional view of treatment effects. The network meta-analysis framework allowed integration of direct and indirect evidence, enabling a comparative ranking of interventions that is not possible through pairwise comparisons alone.

## 5 Conclusion

This network meta-analysis found that, for chronic non-specific low back pain, combined interventions—particularly balneotherapy + general care, aquatic exercise + general care, and balneotherapy + land-based exercise—generally ranked highest for improving pain and disability. Among single-component therapies, aquatic exercise and balneotherapy tended to rank above land-based exercise and general care, though differences were small and may not be clinically meaningful. General care was among the lowest active treatments, while blank control groups ranked worst overall. Besides, disability outcomes placed balneotherapy and aquatic exercise alone slightly higher, suggesting potential added value for functional recovery. These findings support hydrotherapeutic and aquatic-based programs—especially when integrated with general care or complementary exercise—as effective CLBP treatment options. Where aquatic access is limited, land-based exercise remains viable, though extra benefit from adding general care may require sufficient intensity and duration.

Given the limited number of trials for some combination nodes, variation in “general care” protocols, and the scarcity of longer-term follow-up, future RCTs should prioritise rigorous intervention specification and clinically informative follow-up. Specifically, trials should (1) use detailed intervention manuals with fidelity monitoring and reporting (dose, progression, and adherence); (2) include follow-up at 3, 6, and 12 months to determine durability; (3) implement outcome-assessor blinding using aquatic-feasible procedures (e.g., centralised/telephone-based assessments); (4) be adequately powered (e.g., ≥200 participants per comparison) to provide precise estimates and enable planned subgroup analyses; and (5) measure and test potential effect modifiers, including baseline symptom severity, psychological factors (e.g., catastrophizing, kinesiophobia), and setting-specific factors (e.g., water temperature/mineral content and treatment context), to identify which patient subgroups benefit most from aquatic- or spa-based approaches. Given very high heterogeneity and low-to-very-low certainty for most comparisons, these findings should be interpreted as preliminary directional evidence rather than actionable treatment recommendations. Results suggest multimodal approaches warrant investigation in future rigorously conducted trials.

## Data Availability

The original contributions presented in the study are included in the article/Supplementary material, further inquiries can be directed to the corresponding authors.
