# Supplementary material for: In water or on land? A network meta-analysis of aquatic and land-based exercise interventions for pain and disability in chronic lower back pain
Source: Front Med (Lausanne). 2026 Feb 2;13:1739263. doi: 10.3389/fmed.2026.1739263 (PMC12907308; doi:10.3389/fmed.2026.1739263)
Supplement: Supplementary file 1 [file Supplementary_file_1.docx]

Supplementary Material

# Supplementary Tables

**Supplementary Table 1** Search Strategy for PubMed

| # | Searches | n |
| --- | --- | --- |
| 1 | Low Back Pain[Mesh] | 29,524 |
| 2 | (((((((Back pain[Title/Abstract]) OR (Lower Back Pain[Title/Abstract])) OR (Low Back Ache[Title/Abstract])) OR (Postural Low Back Pain[Title/Abstract])) OR (Low Back Pain, Posterior Compartment[Title/Abstract])) OR (Low Back Pain, Recurrent[Title/Abstract])) OR (Recurrent Low Back Pain[Title/Abstract])) OR (Mechanical Low Back Pain[Title/Abstract]) | 68,538 |
| 3 | #1 OR #2 | 74,521 |
| 4 | Hydrotherapy[Mesh] | 21,534 |
| 5 | ((((((((((Hydrotherapies[Title/Abstract]) OR (Whirlpool Bath[Title/Abstract])) OR (balneotherapy[Title/Abstract])) OR (Pool Therapy[Title/Abstract])) OR (aquatic therapy[Title/Abstract])) OR (pool exercise[Title/Abstract])) OR (water-based[Title/Abstract])) OR (aquatic exercise[Title/Abstract])) OR (water training[Title/Abstract])) OR (Water Exercise Therapy[Title/Abstract])) | 8,047 |
| 6 | #4 OR #5 | 29,295 |
| 7 | #3 AND #6 | 161 |

**Supplementary Table 2** Search Strategy for Embase

| # | Searches | n |
| --- | --- | --- |
| 1 | 'low back pain'/exp | 86,146 |
| 2 | 'back pain':ti,ab,kw OR 'lower back pain':ti,ab,kw OR 'low back ache':ti,ab,kw OR 'postural low back pain':ti,ab,kw OR 'low back pain, posterior compartment':ti,ab,kw OR 'low back pain, recurrent':ti,ab,kw OR 'recurrent low back pain':ti,ab,kw OR 'mechanical low back pain':ti,ab,kw | 102,213 |
| 3 | #1 OR #2 | 133,488 |
| 4 | 'hydrotherapy'/exp | 5,822 |
| 5 | hydrotherapies:ti,ab,kw OR 'whirlpool bath':ti,ab,kw OR balneotherapy:ti,ab,kw OR 'pool therapy':ti,ab,kw OR 'aquatic therapy':ti,ab,kw OR 'pool exercise':ti,ab,kw OR 'water based':ti,ab,kw OR 'aquatic exercise':ti,ab,kw OR 'water training':ti,ab,kw OR 'water exercise therapy':ti,ab,kw | 9,929 |
| 6 | #4 OR #5 | 15,125 |
| 7 | #3 AND #6 | 373 |

**Supplementary Table 3.** Search Strategy for Cochrane Library

| # | Searches | n |
| --- | --- | --- |
| 1 | MeSH descriptor: [Low Back Pain] explode all trees | 6,379 |
| 2 | (Back pain):ti,ab,kw OR (Lower Back Pain):ti,ab,kw OR (Low Back Ache):ti,ab,kw OR (Postural Low Back Pain):ti,ab,kw OR (Low Back Pain, Posterior Compartment):ti,ab,kw OR (Low Back Pain, Recurrent):ti,ab,kw OR (Recurrent Low Back Pain):ti,ab,kw OR (Mechanical Low Back Pain):ti,ab,kw | 26,392 |
| 3 | #1 OR #2 | 25,154 |
| 4 | MeSH descriptor: [Hydrotherapy] explode all trees | 1,936 |
| 5 | (Hydrotherapies):ti,ab,kw OR (Whirlpool Bath):ti,ab,kw OR (balneotherapy):ti,ab,kw OR (Pool Therapy):ti,ab,kw OR (aquatic therapy):ti,ab,kw OR (pool exercise):ti,ab,kw OR (water-based):ti,ab,kw OR (aquatic exercise):ti,ab,kw OR (water training):ti,ab,kw OR (Water Exercise Therapy):ti,ab,kw | 7,330 |
| 6 | #4 OR #5 | 8,442 |
| 7 | #3 AND #6 | 277 |

**Supplementary Table 4.** Study-specific standardized mean differences (Hedges’ g) with 95% CIs for all within-study direct comparisons at post-treatment for pain reduction for CLBP. Positive values indicate improvement.

| Study | TreatA | TreatB | SMD | 95%CI lower | 95%CI upper |
| --- | --- | --- | --- | --- | --- |
| Abadi 2019 | Blank Control | Aquatic Exercise | 1.15 | 0.48 | 1.82 |
| Alikhajeh 2020 | Blank Control | Aquatic Exercise | 4.22 | 2.75 | 5.69 |
| Bello 2010 | Land-Based Exercise | Aquatic Exercise | 0 | -1.04 | 1.04 |
| Constant 1997 | General Care | Balneotherapy + General Care | 0.82 | 0.46 | 1.19 |
| Constant 1998 | General Care | Balneotherapy + General Care | 1.07 | 0.78 | 1.35 |
| Demirel 2008 | Land-Based Exercise | Balneotherapy + Land-Based Exercise | 0.1 | -0.48 | 0.68 |
| Dilekci 2020 | General Care | Balneotherapy + General Care | 2.25 | 1.94 | 2.56 |
| Dogan 2011 | General Care | Balneotherapy + General Care | 0.62 | 0.1 | 1.14 |
| Dundar 2009 | Land-Based Exercise | Aquatic Exercise | 0.06 | -0.42 | 0.54 |
| Gati 2018 | General Care | Balneotherapy + General Care | 0.89 | 0.49 | 1.29 |
| Guillemin 1994 | General Care | Balneotherapy + General Care | 2.26 | 1.77 | 2.76 |
| Gunsoo 2011 | Blank Control | Aquatic Exercise | 0.8 | -0.1 | 1.7 |
| Kesiktas 2012 | Balneotherapy + Land-Based Exercise | Land-Based Exercise + General Care | -0.13 | -0.63 | 0.37 |
| Konard 1992 | Blank Control | Balneotherapy | 0.9 | 0.45 | 1.34 |
| Mirmoezzi 2021 | Blank Control | Aquatic Exercise | 1.73 | 0.87 | 2.59 |
| Onat 2014 | General Care | Balneotherapy + General Care | 2.59 | 2 | 3.19 |
| Peng 2022 | General Care | Aquatic Exercise | 0.22 | -0.14 | 0.59 |
| Pires 2015 | Aquatic Exercise | Aquatic Exercise + General Care | 0.39 | -0.11 | 0.88 |
| Sjogren 1997 | Land-Based Exercise | Aquatic Exercise | 0.22 | -0.28 | 0.72 |
| Takinaci 2019 | Balneotherapy | Balneotherapy + Land-Based Exercise | 0.02 | -0.53 | 0.56 |
| Yozbatiran 2004 | Land-Based Exercise | Aquatic Exercise | 0.4 | -0.31 | 1.1 |
| Yucesoy 2021 | Land-Based Exercise | Balneotherapy + Land-Based Exercise | 0.74 | 0.28 | 1.21 |
| Yalfani 2020 | Land-Based Exercise | Aquatic Exercise | -0.07 | -0.84 | 0.7 |
| Bayattork 2022 | Land-Based Exercise | Aquatic Exercise | -0.13 | -0.74 | 0.47 |
| Bayattork 2022 | Land-Based Exercise | General Care | -0.77 | -1.4 | -0.13 |
| Bayattork 2022 | General Care | Aquatic Exercise | 0.69 | 0.06 | 1.31 |

**Supplementary Table 5.** Study-specific standardized mean differences (Hedges’ g) with 95% CIs for all within-study direct comparisons at post-treatment for disability reduction for CLBP. Positive values indicate improvement.

| Study | TreatA | TreatB | SMD | 95%CI lower | 95%CI upper |
| --- | --- | --- | --- | --- | --- |
| Abadi 2019 | Blank Control | Aquatic Exercise | 1.78 | 1.05 | 2.52 |
| Alikhajeh 2020 | Blank Control | Aquatic Exercise | 4.23 | 2.76 | 5.69 |
| Ansari 2021 | Blank Control | Aquatic Exercise | 2.61 | 1.41 | 3.8 |
| Constant 1997 | General Care | Balneotherapy + General Care | 0.93 | 0.56 | 1.29 |
| Constant 1998 | General Care | Balneotherapy + General Care | 0.71 | 0.44 | 0.99 |
| Demirel 2008 | Land-Based Exercise | Balneotherapy + Land-Based Exercise | 0.23 | -0.36 | 0.81 |
| Dilekci 2020 | General Care | Balneotherapy + General Care | 2.24 | 1.93 | 2.55 |
| Dogan 2011 | General Care | Balneotherapy + General Care | 0.79 | 0.27 | 1.32 |
| Dundar 2009 | Land-Based Exercise | Aquatic Exercise | 1.1 | 0.58 | 1.61 |
| Gati 2018 | General Care | Balneotherapy + General Care | 0.61 | 0.22 | 1 |
| Kesiktas 2012 | Balneotherapy + Land-Based Exercise | Land-Based Exercise + General Care | -0.34 | -0.85 | 0.16 |
| Mirmoezzi 2021 | Blank Control | Aquatic Exercise | 0.69 | -0.06 | 1.43 |
| Nemcic 2013 | Land-Based Exercise | Aquatic Exercise | -0.11 | -0.57 | 0.35 |
| Onat 2014 | General Care | Balneotherapy + General Care | 1.27 | 0.79 | 1.75 |
| Peng 2022 | General Care | Aquatic Exercise | 0.37 | 0 | 0.74 |
| Pires 2015 | Aquatic Exercise | Aquatic Exercise + General Care | 0.24 | -0.25 | 0.74 |
| Sjogren 1997 | Land-Based Exercise | Aquatic Exercise | 0.05 | -0.45 | 0.55 |
| Takinaci 2019 | Balneotherapy | Balneotherapy + Land-Based Exercise | -0.42 | -0.97 | 0.13 |
| Yozbatiran 2004 | Land-Based Exercise | Aquatic Exercise | 0.12 | -0.57 | 0.82 |
| Yucesoy 2021 | Land-Based Exercise | Balneotherapy + Land-Based Exercise | 0.22 | -0.24 | 0.67 |
| Yalfani 2020 | Land-Based Exercise | Aquatic Exercise | -0.26 | -1.03 | 0.52 |

**Supplementary Table 6.** CINeMA domain ratings and overall certainty by comparison — pain intensity

| Comparison | Number of studies | Within-study bias | Reporting bias | Indirectness | Imprecision | Heterogeneity | Incoherence | Confidence rating | Reason(s) for downgrading |
| --- | --- | --- | --- | --- | --- | --- | --- | --- | --- |
| Mixed evidence | | | | | | | | | |
| Aquatic Exercise:Aquatic Exercise + General Care | 1 | Some concerns | Low risk | No concerns | Major concerns | No concerns | No concerns | Very low | Within-study bias,Imprecision |
| Aquatic Exercise:Blank Control | 4 | Major concerns | Low risk | No concerns | No concerns | No concerns | No concerns | Low | Within-study bias |
| Aquatic Exercise:General Care | 2 | Some concerns | Low risk | No concerns | Some concerns | Some concerns | No concerns | Very low | Within-study bias,Imprecision,Heterogeneity |
| Aquatic Exercise:Land-Based Exercise | 6 | Major concerns | Low risk | No concerns | Some concerns | Some concerns | No concerns | Very low | Within-study bias,Imprecision,Heterogeneity |
| Balneotherapy:Balneotherapy + Land-Based Exercise | 1 | Major concerns | Low risk | No concerns | Major concerns | No concerns | No concerns | Very low | Within-study bias,Imprecision |
| Balneotherapy:Blank Control | 1 | Major concerns | Low risk | No concerns | No concerns | Some concerns | No concerns | Very low | Within-study bias,Heterogeneity |
| Balneotherapy + General Care:General Care | 7 | Some concerns | Low risk | No concerns | No concerns | No concerns | No concerns | Moderate | Within-study bias |
| Balneotherapy + Land-Based Exercise:Land-Based Exercise | 2 | Some concerns | Low risk | No concerns | Major concerns | No concerns | No concerns | Very low | Within-study bias,Imprecision |
| Balneotherapy + Land-Based Exercise:Land-Based Exercise + General Care | 1 | Some concerns | Low risk | No concerns | Major concerns | No concerns | No concerns | Very low | Within-study bias,Imprecision |
| General Care:Land-Based Exercise | 1 | Some concerns | Low risk | No concerns | Major concerns | No concerns | No concerns | Very low | Within-study bias,Imprecision |
| Indirect evidence | | | | | | | | | |
| Aquatic Exercise:Balneotherapy | 0 | Major concerns | Low risk | No concerns | Major concerns | No concerns | No concerns | Very low | Within-study bias,Imprecision |
| Aquatic Exercise:Balneotherapy + General Care | 0 | Some concerns | Low risk | No concerns | Some concerns | Some concerns | No concerns | Very low | Within-study bias,Imprecision,Heterogeneity |
| Aquatic Exercise:Balneotherapy + Land-Based Exercise | 0 | Some concerns | Low risk | No concerns | Major concerns | No concerns | No concerns | Very low | Within-study bias,Imprecision |
| Aquatic Exercise:Land-Based Exercise + General Care | 0 | Some concerns | Low risk | No concerns | Major concerns | No concerns | No concerns | Very low | Within-study bias,Imprecision |
| Aquatic Exercise + General Care:Balneotherapy | 0 | Major concerns | Low risk | No concerns | Major concerns | No concerns | No concerns | Very low | Within-study bias,Imprecision |
| Aquatic Exercise + General Care:Balneotherapy + General Care | 0 | Some concerns | Low risk | No concerns | Major concerns | No concerns | No concerns | Very low | Within-study bias,Imprecision |
| Aquatic Exercise + General Care:Balneotherapy + Land-Based Exercise | 0 | Some concerns | Low risk | No concerns | Major concerns | No concerns | No concerns | Very low | Within-study bias,Imprecision |
| Aquatic Exercise + General Care:Blank Control | 0 | Major concerns | Low risk | No concerns | No concerns | Some concerns | No concerns | Very low | Within-study bias,Heterogeneity |
| Aquatic Exercise + General Care:General Care | 0 | Some concerns | Low risk | No concerns | Major concerns | No concerns | No concerns | Very low | Within-study bias,Imprecision |
| Aquatic Exercise + General Care:Land-Based Exercise | 0 | Some concerns | Low risk | No concerns | Major concerns | No concerns | No concerns | Very low | Within-study bias,Imprecision |
| Aquatic Exercise + General Care:Land-Based Exercise + General Care | 0 | Some concerns | Low risk | No concerns | Major concerns | No concerns | No concerns | Very low | Within-study bias,Imprecision |
| Balneotherapy:Balneotherapy + General Care | 0 | Some concerns | Low risk | No concerns | Some concerns | Some concerns | No concerns | Very low | Within-study bias,Imprecision,Heterogeneity |
| Balneotherapy:General Care | 0 | Major concerns | Low risk | No concerns | Major concerns | No concerns | No concerns | Very low | Within-study bias,Imprecision |
| Balneotherapy:Land-Based Exercise | 0 | Major concerns | Low risk | No concerns | Major concerns | No concerns | No concerns | Very low | Within-study bias,Imprecision |
| Balneotherapy:Land-Based Exercise + General Care | 0 | Major concerns | Low risk | No concerns | Major concerns | No concerns | No concerns | Very low | Within-study bias,Imprecision |
| Balneotherapy + General Care:Balneotherapy + Land-Based Exercise | 0 | Some concerns | Low risk | No concerns | Major concerns | No concerns | No concerns | Very low | Within-study bias,Imprecision |
| Balneotherapy + General Care:Blank Control | 0 | Some concerns | Low risk | No concerns | No concerns | No concerns | No concerns | Moderate | Within-study bias |
| Balneotherapy + General Care:Land-Based Exercise | 0 | Some concerns | Low risk | No concerns | Some concerns | Some concerns | No concerns | Very low | Within-study bias,Imprecision,Heterogeneity |
| Balneotherapy + General Care:Land-Based Exercise + General Care | 0 | Some concerns | Low risk | No concerns | Major concerns | No concerns | No concerns | Very low | Within-study bias,Imprecision |
| Balneotherapy + Land-Based Exercise:Blank Control | 0 | Major concerns | Low risk | No concerns | No concerns | Some concerns | No concerns | Very low | Within-study bias,Heterogeneity |
| Balneotherapy + Land-Based Exercise:General Care | 0 | Some concerns | Low risk | No concerns | Major concerns | No concerns | No concerns | Very low | Within-study bias,Imprecision |
| Blank Control:General Care | 0 | Major concerns | Low risk | No concerns | Some concerns | Some concerns | No concerns | Very low | Within-study bias,Imprecision,Heterogeneity |
| Blank Control:Land-Based Exercise | 0 | Major concerns | Low risk | No concerns | No concerns | Some concerns | No concerns | Very low | Within-study bias,Heterogeneity |
| Blank Control:Land-Based Exercise + General Care | 0 | Some concerns | Low risk | No concerns | Some concerns | Some concerns | No concerns | Very low | Within-study bias,Imprecision,Heterogeneity |
| General Care:Land-Based Exercise + General Care | 0 | Some concerns | Low risk | No concerns | Major concerns | No concerns | No concerns | Very low | Within-study bias,Imprecision |
| Land-Based Exercise:Land-Based Exercise + General Care | 0 | Some concerns | Low risk | No concerns | Major concerns | No concerns | No concerns | Very low | Within-study bias,Imprecision |

**Supplementary Table 7.** CINeMA domain ratings and overall certainty by comparison — disability

| Comparison | Number of studies | Within-study bias | Reporting bias | Indirectness | Imprecision | Heterogeneity | Incoherence | Confidence rating | Reason(s) for downgrading |
| --- | --- | --- | --- | --- | --- | --- | --- | --- | --- |
| Mixed evidence | | | | | | | | | |
| Aquatic Exercise:Aquatic Exercise + General Care | 1 | Some concerns | Low risk | No concerns | Major concerns | No concerns | Major concerns | Very low | Within-study bias,Imprecision,Incoherence |
| Aquatic Exercise:Blank Control | 4 | Major concerns | Low risk | No concerns | No concerns | No concerns | Major concerns | Very low | Within-study bias,Incoherence |
| Aquatic Exercise:General Care | 1 | Some concerns | Low risk | No concerns | Major concerns | No concerns | Major concerns | Very low | Within-study bias,Imprecision,Incoherence |
| Aquatic Exercise:Land-Based Exercise | 5 | Major concerns | Low risk | No concerns | Some concerns | Some concerns | Major concerns | Very low | Within-study bias,Imprecision,Heterogeneity,Incoherence |
| Balneotherapy:Balneotherapy + Land-Based Exercise | 1 | Major concerns | Low risk | No concerns | Major concerns | No concerns | Major concerns | Very low | Within-study bias,Imprecision,Incoherence |
| Balneotherapy + General Care:General Care | 6 | Some concerns | Low risk | No concerns | No concerns | Some concerns | Major concerns | Very low | Within-study bias,Heterogeneity,Incoherence |
| Balneotherapy + Land-Based Exercise:Land-Based Exercise | 2 | Some concerns | Low risk | No concerns | Major concerns | No concerns | Major concerns | Very low | Within-study bias,Imprecision,Incoherence |
| Balneotherapy + Land-Based Exercise:Land-Based Exercise + General Care | 1 | Some concerns | Low risk | No concerns | Major concerns | No concerns | Major concerns | Very low | Within-study bias,Imprecision,Incoherence |
| Indirect evidence | | | | | | | | | |
| Aquatic Exercise:Balneotherapy | 0 | Major concerns | Low risk | No concerns | Major concerns | No concerns | Major concerns | Very low | Within-study bias,Imprecision,Incoherence |
| Aquatic Exercise:Balneotherapy + General Care | 0 | Some concerns | Low risk | No concerns | Major concerns | No concerns | Major concerns | Very low | Within-study bias,Imprecision,Incoherence |
| Aquatic Exercise:Balneotherapy + Land-Based Exercise | 0 | Some concerns | Low risk | No concerns | Major concerns | No concerns | Major concerns | Very low | Within-study bias,Imprecision,Incoherence |
| Aquatic Exercise:Land-Based Exercise + General Care | 0 | Some concerns | Low risk | No concerns | Major concerns | No concerns | Major concerns | Very low | Within-study bias,Imprecision,Incoherence |
| Aquatic Exercise + General Care:Balneotherapy | 0 | Some concerns | Low risk | No concerns | Major concerns | No concerns | Major concerns | Very low | Within-study bias,Imprecision,Incoherence |
| Aquatic Exercise + General Care:Balneotherapy + General Care | 0 | Some concerns | Low risk | No concerns | Major concerns | No concerns | Major concerns | Very low | Within-study bias,Imprecision,Incoherence |
| Aquatic Exercise + General Care:Balneotherapy + Land-Based Exercise | 0 | Some concerns | Low risk | No concerns | Major concerns | No concerns | Major concerns | Very low | Within-study bias,Imprecision,Incoherence |
| Aquatic Exercise + General Care:Blank Control | 0 | Major concerns | Low risk | No concerns | No concerns | No concerns | Major concerns | Very low | Within-study bias,Incoherence |
| Aquatic Exercise + General Care:General Care | 0 | Some concerns | Low risk | No concerns | Major concerns | No concerns | Major concerns | Very low | Within-study bias,Imprecision,Incoherence |
| Aquatic Exercise + General Care:Land-Based Exercise | 0 | Some concerns | Low risk | No concerns | Major concerns | No concerns | Major concerns | Very low | Within-study bias,Imprecision,Incoherence |
| Aquatic Exercise + General Care:Land-Based Exercise + General Care | 0 | Some concerns | Low risk | No concerns | Major concerns | No concerns | Major concerns | Very low | Within-study bias,Imprecision,Incoherence |
| Balneotherapy:Balneotherapy + General Care | 0 | Some concerns | Low risk | No concerns | Major concerns | No concerns | Major concerns | Very low | Within-study bias,Imprecision,Incoherence |
| Balneotherapy:Blank Control | 0 | Major concerns | Low risk | No concerns | No concerns | Some concerns | Major concerns | Very low | Within-study bias,Heterogeneity,Incoherence |
| Balneotherapy:General Care | 0 | Some concerns | Low risk | No concerns | Major concerns | No concerns | Major concerns | Very low | Within-study bias,Imprecision,Incoherence |
| Balneotherapy:Land-Based Exercise | 0 | Major concerns | Low risk | No concerns | Major concerns | No concerns | Major concerns | Very low | Within-study bias,Imprecision,Incoherence |
| Balneotherapy:Land-Based Exercise + General Care | 0 | Major concerns | Low risk | No concerns | Major concerns | No concerns | Major concerns | Very low | Within-study bias,Imprecision,Incoherence |
| Balneotherapy + General Care:Balneotherapy + Land-Based Exercise | 0 | Some concerns | Low risk | No concerns | Major concerns | No concerns | Major concerns | Very low | Within-study bias,Imprecision,Incoherence |
| Balneotherapy + General Care:Blank Control | 0 | Some concerns | Low risk | No concerns | No concerns | No concerns | Major concerns | Very low | Within-study bias,Incoherence |
| Balneotherapy + General Care:Land-Based Exercise | 0 | Some concerns | Low risk | No concerns | Major concerns | No concerns | Major concerns | Very low | Within-study bias,Imprecision,Incoherence |
| Balneotherapy + General Care:Land-Based Exercise + General Care | 0 | Some concerns | Low risk | No concerns | Major concerns | No concerns | Major concerns | Very low | Within-study bias,Imprecision,Incoherence |
| Balneotherapy + Land-Based Exercise:Blank Control | 0 | Major concerns | Low risk | No concerns | No concerns | Some concerns | Major concerns | Very low | Within-study bias,Heterogeneity,Incoherence |
| Balneotherapy + Land-Based Exercise:General Care | 0 | Some concerns | Low risk | No concerns | Major concerns | No concerns | Major concerns | Very low | Within-study bias,Imprecision,Incoherence |
| Blank Control:General Care | 0 | Major concerns | Low risk | No concerns | No concerns | Major concerns | Major concerns | Very low | Within-study bias,Heterogeneity,Incoherence |
| Blank Control:Land-Based Exercise | 0 | Major concerns | Low risk | No concerns | No concerns | No concerns | Major concerns | Very low | Within-study bias,Incoherence |
| Blank Control:Land-Based Exercise + General Care | 0 | Some concerns | Low risk | No concerns | Some concerns | Some concerns | Major concerns | Very low | Within-study bias,Imprecision,Heterogeneity,Incoherence |
| General Care:Land-Based Exercise | 0 | Some concerns | Low risk | No concerns | Major concerns | No concerns | Major concerns | Very low | Within-study bias,Imprecision,Incoherence |
| General Care:Land-Based Exercise + General Care | 0 | Some concerns | Low risk | No concerns | Major concerns | No concerns | Major concerns | Very low | Within-study bias,Imprecision,Incoherence |
| Land-Based Exercise:Land-Based Exercise + General Care | 0 | Some concerns | Low risk | No concerns | Major concerns | No concerns | Major concerns | Very low | Within-study bias,Imprecision,Incoherence |

**Supplementary Table 8.** Assessment of inconsistency in the network meta-analysis— pain

| **Level** | **Method** | **Contrast / Loop** | **Inconsistency statistic** | **P** |
| --- | --- | --- | --- | --- |
| Global | Design-by-treatment interaction (Wald) | Overall network | χ²(3)=1.34 | 0.72 |
| Local | Node-splitting (side-splitting) | A vs D | Diff=1.177 | 0.32 |
| Local | Node-splitting (side-splitting) | A vs E | Diff=−1.176 | 0.32 |
| Local | Node-splitting (side-splitting) | B vs C | Diff=−0.794 | 0.43 |
| Local | Node-splitting (side-splitting) | B vs D | Diff=−0.475 | 0.65 |
| Local | Node-splitting (side-splitting) | B vs G | Diff=1.177 | 0.32 |
| Local | Node-splitting (side-splitting) | C vs D | Diff=−0.918 | 0.54 |
| Local | Node-splitting (side-splitting) | C vs F | Diff=3.548 | 0.99 |
| Local | Node-splitting (side-splitting) | D vs I | Diff=3.527 | 0.99 |
| Local | Node-splitting (side-splitting) | E vs G | Diff=−1.177 | 0.32 |
| Local | Node-splitting (side-splitting) | G vs H | Diff=3.227 | 0.99 |
| Local | Loop-specific approach | B–C–D loop | IF=0.625 | 0.11 |

Note: For some contrasts, indirect evidence was sparse/absent, resulting in unstable indirect estimates (very large standard errors) in node-splitting; therefore, these local inconsistency checks should be interpreted as non-informative and are reported mainly for transparency. A = Blank Control; B = Land-Based Exercise; C = General Care; D = Aquatic Exercise; E = Balneotherapy; F = Balneotherapy + General Care; G = Balneotherapy + Land-Based Exercise; H = Land-Based Exercise + General Care; I = Aquatic Exercise + General Care.

**Supplementary Table 9.** Assessment of inconsistency in the network meta-analysis— disability

| **Level** | **Method** | **Contrast / Loop** | **Inconsistency statistic** | **P** |
| --- | --- | --- | --- | --- |
| Global | Design-by-treatment interaction (Wald) | Overall network | χ²(1)=0.25 | 0.62 |
| Local | Node-splitting (side-splitting) | A vs D | Diff=2.064 | 0.98 |
| Local | Node-splitting (side-splitting) | B vs D | Diff=−2.455 | 0.99 |
| Local | Node-splitting (side-splitting) | B vs G | Diff=2.440 | 0.99 |
| Local | Node-splitting (side-splitting) | C vs D | Diff=−2.284 | 0.98 |
| Local | Node-splitting (side-splitting) | C vs F | Diff=4.451 | 0.98 |
| Local | Node-splitting (side-splitting) | D vs I | Diff=4.306 | 0.99 |
| Local | Node-splitting (side-splitting) | E vs G | Diff=−4.522 | 0.99 |

Note: For some contrasts, indirect evidence was sparse/absent, resulting in unstable indirect estimates (very large standard errors) in node-splitting; therefore, these local inconsistency checks should be interpreted as non-informative and are reported mainly for transparency. For disability, no closed loops were present, so loop-specific inconsistency factors were not applicable. A = Blank Control; B = Land-Based Exercise; C = General Care; D = Aquatic Exercise; E = Balneotherapy; F = Balneotherapy + General Care; G = Balneotherapy + Land-Based Exercise; H = Land-Based Exercise + General Care; I = Aquatic Exercise + General Care.

# Supplementary Figures

Leave-one-out sensitivity analyses; dots show the network estimate after removing one study, bars are 95% CIs, dashed line denotes the null. Effects remained directionally consistent; occasional marginal significance or wider CIs did not change conclusions.


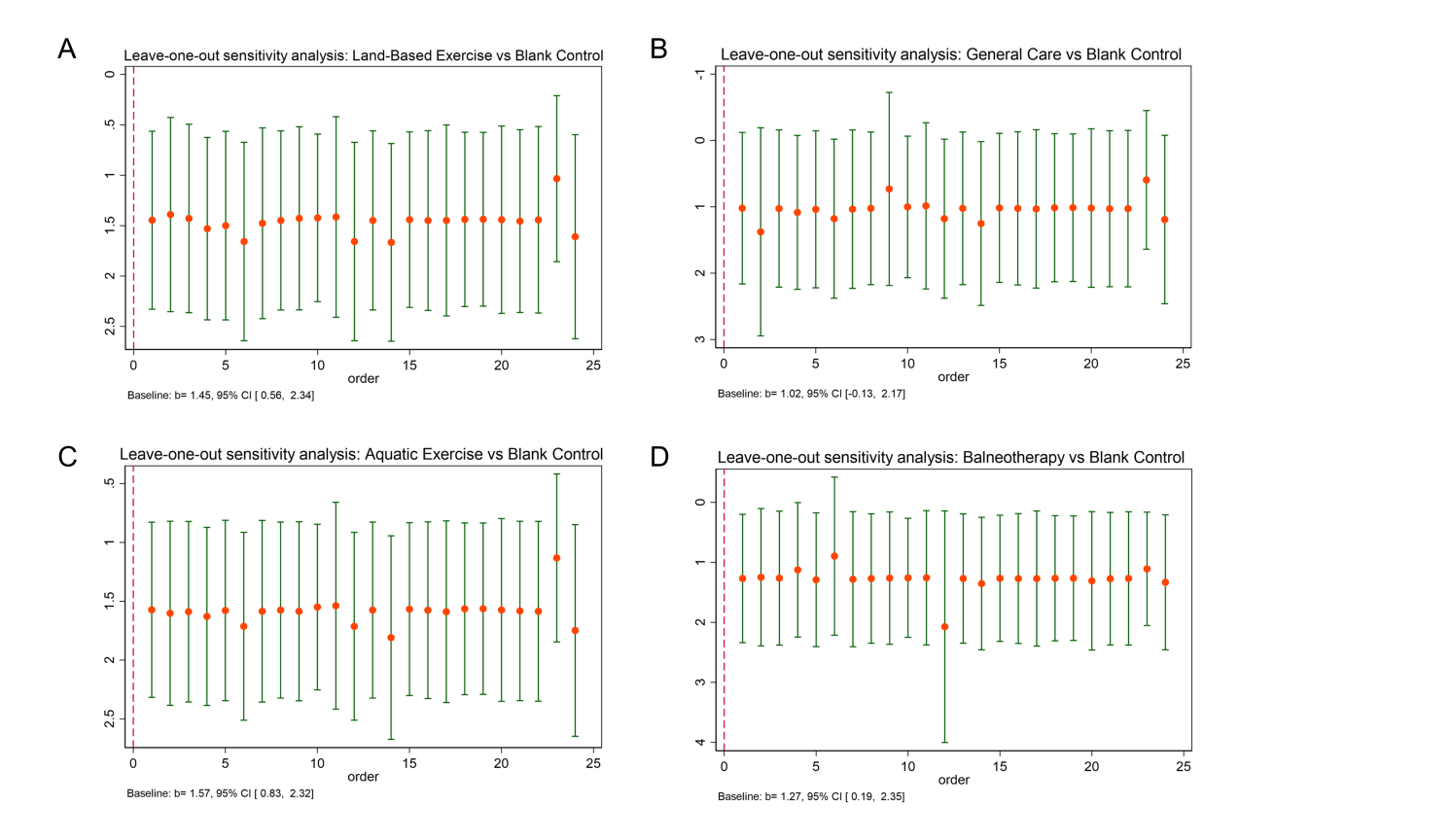


**Supplementary Figure 1.** Leave-one-out (LOO) influence plots for pain intensity: single-component interventions vs Blank Control.

Panels: A Land-Based Exercise vs Blank Control; B General Care vs Blank Control; C Aquatic Exercise vs Blank Control; D Balneotherapy vs Blank Control.


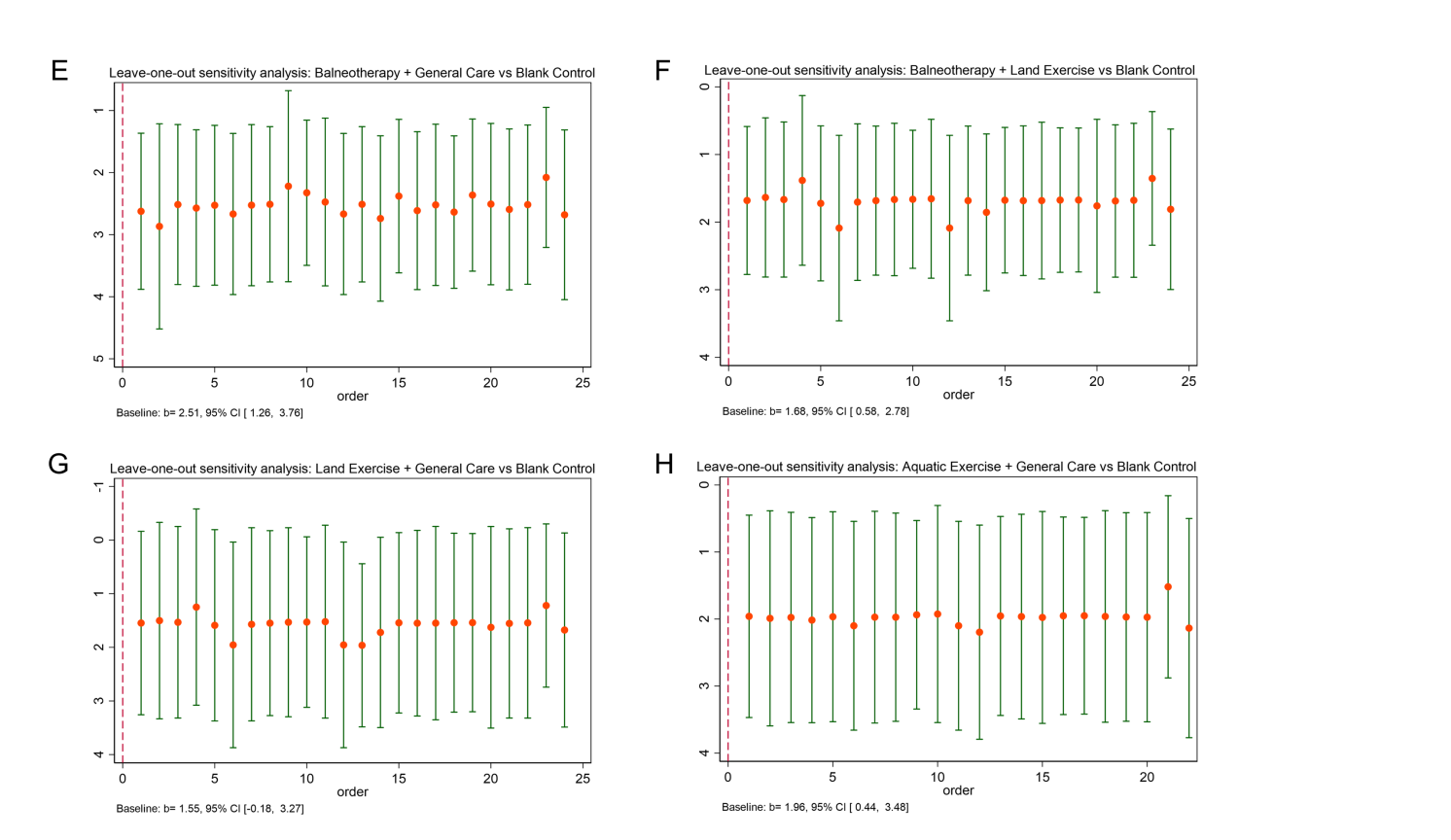


**Supplementary Figure 2.** Leave-one-out (LOO) influence plots for pain intensity: combined interventions vs Blank Control.

Panels: E Balneotherapy + General Care vs Blank Control; F Balneotherapy + Land-Based Exercise vs Blank Control; G Land-Based Exercise + General Care vs Blank Control; H Aquatic Exercise + General Care vs Blank Control.


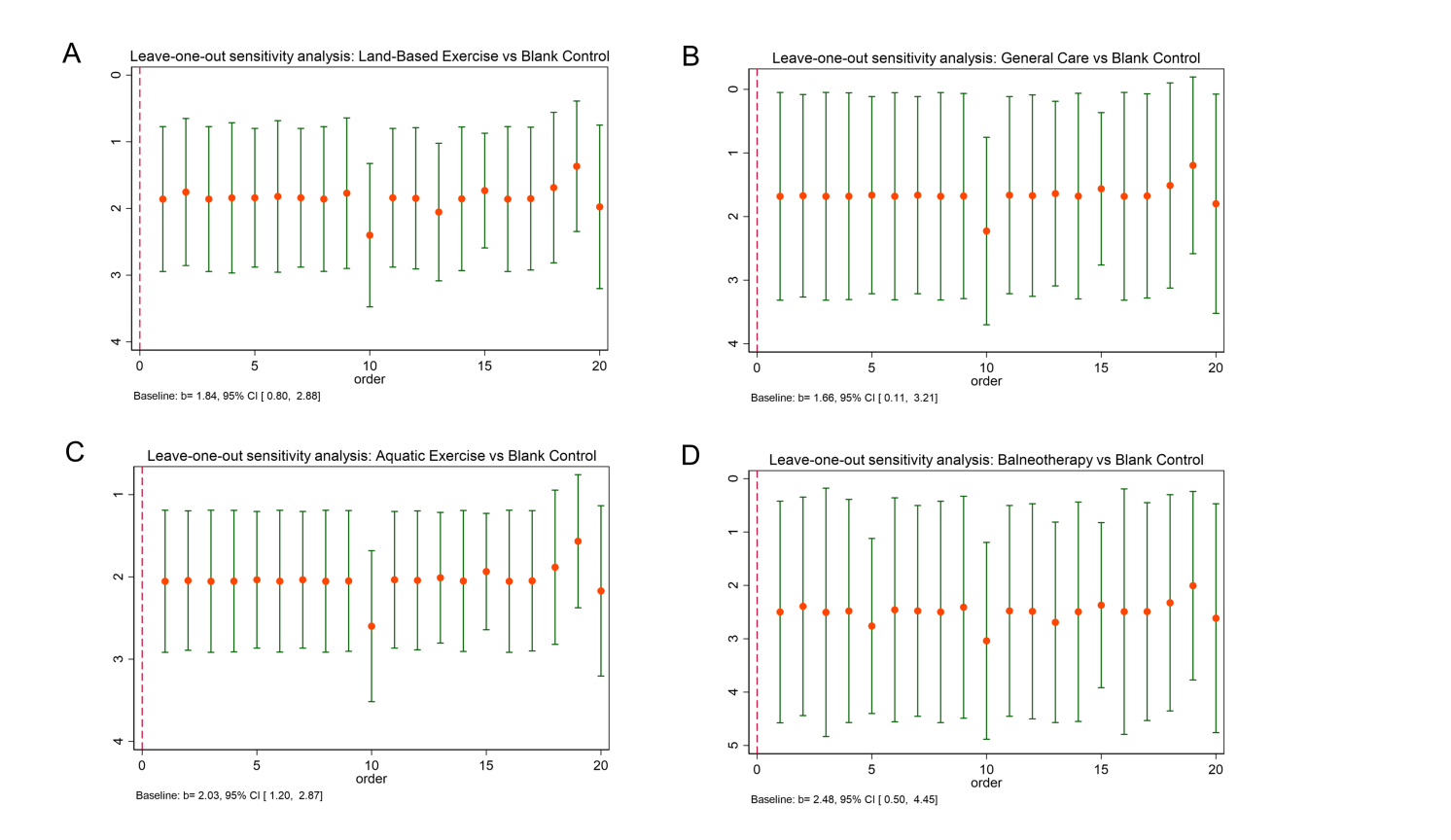


**Supplementary Figure 3.** Leave-one-out (LOO) influence plots for disability: single-component interventions vs Blank Control.

Panels: A Land-Based Exercise vs Blank Control; B General Care vs Blank Control; C Aquatic Exercise vs Blank Control; D Balneotherapy vs Blank Control.


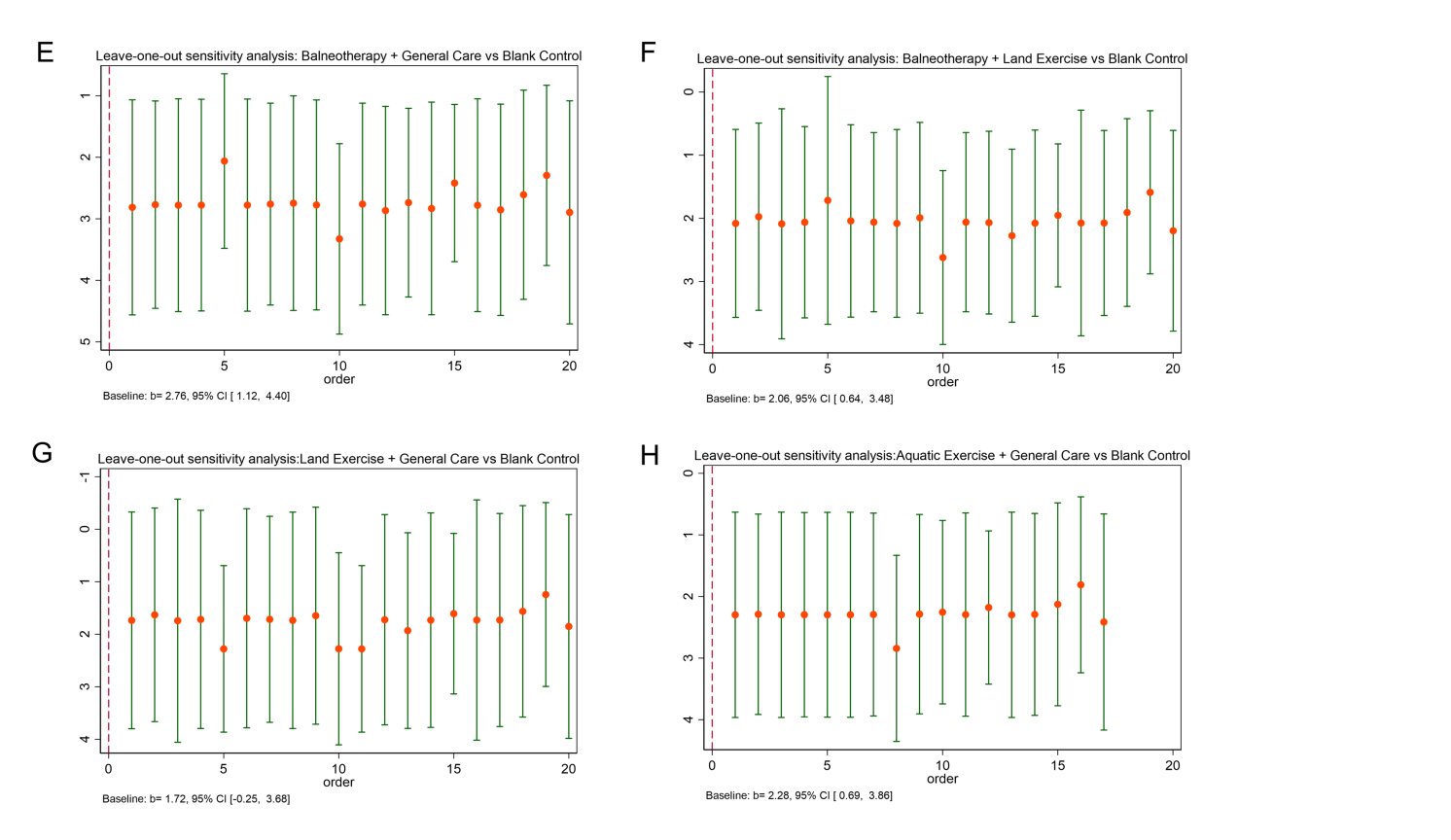


**Supplementary Figure 4.** Leave-one-out (LOO) influence plots for disability: combined interventions vs Blank Control.

Panels: E Balneotherapy + General Care vs Blank Control; F Balneotherapy + Land-Based Exercise vs Blank Control; G Land-Based Exercise + General Care vs Blank Control; H Aquatic Exercise + General Care vs Blank Control.
